# Galectin-7 reprograms skin carcinogenesis by fostering innate immune evasive programs

**DOI:** 10.1038/s41418-022-01108-7

**Published:** 2023-01-24

**Authors:** Nicolás A. Pinto, Martín C. Abba, Lorena Laporte, Juan M. Pérez Sáez, Ada G. Blidner, Nicolás I. Torres, Rosa M. Morales, Sabrina G. Gatto, Camila A. Bach, Florencia Veigas, Hernán J. García Rivello, Peng Song, Jane H. Frederiksen, Lene Juel Rasmussen, Francoise Poirier, Diego O. Croci, Victoria Sundblad, Gabriel A. Rabinovich, Juan P. Cerliani

**Affiliations:** 1 Laboratorio de Glicomedicina, Instituto de Biología y Medicina Experimental (IBYME), Consejo Nacional de Investigaciones Científicas y Técnicas (CONICET), C1428 Buenos Aires, Argentina; 2 Centro de Investigaciones Inmunológicas Básicas y Aplicadas (CINIBA), Facultad de Ciencias Médicas, Universidad Nacional de La Plata, C1900 La Plata, Argentina; 3 Universidad Argentina de la Empresa (UADE). Instituto de Tecnología (INTEC), C1073 Buenos Aires, Argentina; 4 Departamento de Dermatología, Hospital Italiano, C1199 Buenos Aires, Argentina; 5 Center for Healthy Aging, Department of Cellular and Molecular Medicine, University of Copenhagen, DK-, 2200 Copenhagen, Denmark; 6 Institut Jacques Monod, UMR CNRS 7592, Paris-Diderot University, Paris, France; 7 Instituto de Histología y Embriología de Mendoza Dr. Mario H. Burgos (IHEM-CONICET), Facultad de Ciencias Exactas y Naturales, Universidad Nacional de Cuyo, C5500 Mendoza, Argentina; 8 Facultad de Ciencias Exactas y Naturales, Universidad de Buenos Aires, C1428 Buenos Aires, Argentina

**Keywords:** Cancer microenvironment, Immune evasion, Glycobiology

## Abstract

Non-melanoma skin cancer (NMSC) has risen dramatically as a result of chronic exposure to sunlight ultraviolet (UV) radiation, climatic changes and clinical conditions associated with immunosuppression. In spite of considerable progress, our understanding of the mechanisms that control NMSC development and their associated molecular and immunological landscapes is still limited. Here we demonstrated a critical role for galectin-7 (Gal-7), a β-galactoside-binding protein preferentially expressed in skin tissue, during NMSC development. Transgenic mice (*Tg46)* overexpressing Gal-7 in keratinocytes showed higher number of papillomas compared to WT mice or mice lacking Gal-7 (*Lgals7*^*−/−*^) when subjected to a skin carcinogenesis protocol, in which tumor initiator 7,12-dimethylbenz[a]anthracene (DMBA) and tumor promoter 12-O-tetradecanoyl-phorbol-13-acetate (TPA) were sequentially administered. RNAseq analysis of *Tg46* tumor lesions revealed a unique profile compatible with cells of the myelomonocytic lineage infiltrating these tumors, an effect that was substantiated by a higher number of CD11b^+^Gr1^+^ cells in tumor-draining lymph nodes. Heightened c-Met activation and Cxcl-1 expression in *Tg46* lesions suggested a contribution of this pathway to the recruitment of these cells. Remarkably, Gal-7 bound to the surface of CD11b^+^Ly6C^hi^Ly6G^lo^ monocytic myeloid cells and enhanced their immunosuppressive activity, as evidenced by increased IL-10 and TGF-β_1_ secretion, and higher T-cell inhibitory activity. In vivo, carcinogen-treated *Lgals7*^*−/−*^ animals adoptively transferred with Gal-7-conditioned monocytic myeloid cells developed higher number of papillomas, whereas depletion of these cells in *Tg46*-treated mice led to reduction in the number of tumors. Finally, human NMSC biopsies showed increased *LGALS7* mRNA and Gal-7 protein expression and displayed transcriptional profiles associated with myeloid programs, accompanied by elevated CXCL1 expression and c-Met activation. Thus, Gal-7 emerges as a critical mediator of skin carcinogenesis and a potential therapeutic target in human NMSC.

## Introduction

Over the past decades non-melanoma skin cancer (NMSC) has risen dramatically worldwide as a result of chronic exposure to sunlight ultraviolet (UV) radiation, climatic changes and clinical conditions associated with immunosuppression [1, 2]. Although surgical excision is the mainstay treatment option for NMSC, standard management strategy for follow-up therapies is still not established [3]. The limited efficacy of anti-NMSC agents [4, 5] urge further exploration of the molecular and immunological landscapes of NMSC aimed at designing selective therapeutic strategies targeting locally-dysregulated pathways.

Upon its continuous exposure to physical and chemical stimuli, the skin takes shelter in the dynamic cross-talk between keratinocytes (KC), melanocytes and immune cells to maintain homeostasis [6]. Eventually, environmental stimuli may induce genetic alterations and activate inflammatory programs that trigger the initial steps of carcinogenesis [2]. This multifaceted process requires the coordinated activation of numerous pathways controlling DNA damage repair, transcriptional regulation and inflammation leading to KC proliferation or death [7].

Galectins, a family of β-galactoside-binding proteins, control tumor progression by influencing different hallmarks of cancer cells including proliferation, angiogenesis, inflammation, immune evasion and metastasis [8] and modulating resistance to anticancer therapies [9]. Whereas galectins control diverse intracellular processes [10], they are also released to the extracellular space where they can cross-link cell surface glycoconjugates and trigger a myriad of cellular processes [11]. Galectin-7 (Gal-7; encoded by the *LGALS7* gene) is preferentially expressed in stratified squamous epithelium of the skin, tongue, esophagus and rectal mucosa [12, 13], where it regulates KC proliferation and differentiation [14]. Originally described as a P53-inducible gene (PIG-1) [15], Gal-7 controls UVB-induced KC apoptosis [13, 16] and influences skin repair, wound healing and inflammation [16–20]. However, despite considerable progress, the precise role of Gal-7 in tumor biology remains poorly understood [21, 22]. Whereas high Gal-7 expression was associated with aggressive behavior, metastasis and tumor recurrence [23–32], inhibitory effects over tumor growth and progression have also been documented [33–37]. In head and neck SCC, expression of Gal-7 correlated with degree of tissue differentiation and keratinization [38]. Up-regulation of *LGALS7* expression has been linked to activation of multiple transcriptional programs involved in tumor survival, metabolism and inflammatory responses [37].

Taken together, the preferential expression of Gal-7 in stratified skin epithelium, and the need of selective immunotherapies for NMSC, prompted us to investigate the role of this endogenous lectin in the pathogenesis of this tumor type. Here, using genetically-engineered mouse models with ablated or tissue-specific reinforced Gal-7 expression, and patient biopsies, we show essential roles of Gal-7 during the initial steps of skin carcinogenesis, highlighting its potential as a therapeutic target in NMSC.

## Materials and methods

### Mice

Mice lacking Gal-7 (*Lgals7*^*−/−*^; C57BL/6) or overexpressing Gal-7 in KCs (*Tg46*) were generated as described [16, 38]. *Mgat5*^*−/−*^ and *C2gnt1*^*−/−*^ (C57BL/6) mice were obtained from Jackson’s Laboratories (Bar Harbour, USA). Age-matched, 6–10-weeks old female mice were used. Mice were bred at the animal facilities of the Institute of Biology and Experimental Medicine (IBYME) according to NIH guidelines. All experimental procedures were approved by the Institutional Committee for the Care and Use of Laboratory Animals. The total number of animals, as well as the number of animals per group (*n* = 5) in each experiment were determined following the guidelines of the mentioned Committee. Age, sex, weight/body condition scores, and health status were considered to establish comparable experimental groups; no randomization method was used to allocate animals to experimental groups.

### Short-term challenge with chemical stressors

Mice were treated topically on the ears or dorsal skin with 200 nmol of 7,12-dimethylbenz[a]anthracene (DMBA) or 20 nmol of 12-O-tetradecanoyl-phorbol-13-acetate (TPA) or exposed to UVB radiation (average dose 3.1 kJ/m^2^ UVB radiation, using a Waldmann UV 236 B (UV6) lamp with 2.62 mW/cm^2^ intensity at 20 cm, for 2 min). Mice not receiving treatment were kept as control. After 16 h, mice were euthanized, and the treated skin was processed (see Supplementary Materials and Methods) for real time qPCR, Western blot, immunohistochemistry and immunofluorescence analysis.

### Chemical skin carcinogenesis and analysis of myeloid cell function

WT, *Lgals7*^*−*/*−*^ and *Tg46* mice were treated topically with 200 nmol DMBA followed by 20 nmol TPA twice a week for about three months, on shaved dorsal back area. For determinations of the role of MDSCs in Gal-7-driven skin carcinogenesis, after the first month, *Lgals7*^*−*/*−*^ mice were injected weekly for the remaining two months with monocytic-myeloid-derived suppressor cells (M-MDSC; 1 × 10^6^ cells/mouse, i.p) previously activated in the presence or absence of rGal-7. *Tg46* animals were injected with the MDSC-depleting anti-DR5 monoclonal antibody (mAb) or isotype control (BioxCell; 150 μg/mouse, weekly). The anti-DR5mAb binds to mouse death receptor-5 (DR5, also known as CD262 and TRAIL-R2), a member of the TNF receptor superfamily, and induces TRAIL-mediated apoptosis of MDSCs [39]. Mice were monitored daily, and tumor volume, incidence and multiplicity were recorded twice a week. At the end of the protocol, animals were euthanized and processed for subsequent studies. No blinding to treatment assignment was used.

### RNA-seq analysis

RNA from mouse skin papillomas (3 pooled tumors for each genotype) was isolated and purified using TRIzol reagent (Invitrogen) and RNeasy mini kit (Qiagen). After checking concentration and integrity (Agilent 2100 Bioanalyzer, Agilent Technologies), RNAs were processed for directional RNA-Seq library construction using the TruSeq RNA Sample Preparation Kit v2 (Illumina) according to the manufacturer protocol. We performed 75-nt paired-end sequencing using an Illumina HiSeq2500 platform at MedGenome Core Facility and obtained ~70 million tags per sample. QC and alignment of the short-read sequences against the human reference genome (mm10) were performed by the ShortRead [40] and Rsubread [41] R/Bioconductor packages respectively. Subsequently, the number of reads mapped to each gene on the basis of the UCSC.mm10. Known Gene database was counted, reported, and annotated using the Genomic Features, Genomic Alignments and org.Mm.eg.db packages. Raw datasets were submitted to NCBI GEO database (GSE165285). To identify genes differentially expressed in WT versus *Lgals7*^*−/−*^ and *Tg46* tumors, we used the DESeq2-test on the basis of the normalized number of reads mapped to each gene [42]. Functional enrichment analysis was performed using the Enrichr resource [43] and Cytoscape´s plugins ClueGo/CluePedia (http://www.cytoscape.org/) based on the list of dysregulated transcripts between samples (*p* < 0.05; FC > ±2). Quantification of the absolute abundance of immune and stromal cell populations was estimated using MCPcounter algorithm (https://github.com/ebecht/MCPcounter) based on RNA-seq data. Data integration and visualization of differentially expressed transcripts were done with R/Bioconductor.

### Production and purification of recombinant Gal-7

Recombinant Gal-7 was obtained from a pGEX-27 plasmid (GeneScript) and purified using a lactosyl-Sepharose column essentially as described for rGal-1 [44]. In brief, *E. coli* BL21 (DE3) cells were transformed with expression plasmids constructed using pET expression systems (Novagen) and production of rGal-7 was induced by the addition of 1 mM isopropyl-D-thiogalactoside. Soluble fractions were obtained for subsequent purification by affinity chromatography on a lactosyl-Sepharose column (Sigma-Aldrich). LPS was carefully removed by Detoxi-Gel endotoxin-removing gel (Pierce), followed by analysis with a Gel Clot Limulus test (<0.5 I U/mg; Cape Code).

For in vitro bone marrow (BM)-MDSC differentiation, 20 μg/ml rGal-7 was used. For rGal-7 binding assays, labeling was performed following manufacturer’s recommendations. Briefly, 6 μl of modifier solution was added to 60 μl rGal-7 (1 mg/ml). Then the solution was incubated with the unbound fluorochrome for 15 min at 4 °C. Thereafter, 7 μl of quenching solution was added. Labeled rGal-7 was aliquoted and stored at 4 °C. Flow cytometry staining was performed with 10 μg/ml of labeled rGal-7. For binding assays 40 μg/ml of labeled rGal-7 was used (see below).

### Flow cytometry

Single cell suspensions from skin, tumor, draining lymph nodes (dLN) and spleen were stained with fluorochrome-conjugated antibodies against CD11b (PA5-79532, ThermoFisher), CD4 (116005, BioLegend), CD8 (100726, BioLegend), CD25 (102011, BioLegend), Foxp3 (320105, BioLegend), Ly6C (128021, BioLegend) or Ly6G (127607, BioLegend) and appropriate isotype controls (BioLegend) for 30 min on ice. Cells were washed and fixed with 1% paraformaldehyde and analyzed on a FACSAriaIIu (BD Biosciences). The frequency of CD4^+^CD25^+^Foxp3^+^ regulatory T cells (Tregs) was determined using the mouse Treg staining kit (eBioscience).

### Immunofluorescence

Tissue samples from ear or dorsal skin were embedded in OCT and frozen at −70 °C. Frozen tissue slides (20 μm) were fixed with 4% paraformaldehyde/PBS for 15 min followed by permeabilization and blocking with 0.2% Triton X-100, 10% FBS/PBS for 30 min at room temperature and stained with primary antibodies anti-Gal-7 (ab138513, Abcam), anti-CD11b (PA5-79532, ThermoFisher), anti-PCNA (sc-25280, Santa Cruz), anti-Gr1 (14-5931-81, eBioscience), Ly6C (128021, BioLegend) or Ly6G (127607, BioLegend) for 16 h at 4 °C. Slides were then washed in PBS and incubated with fluorochrome-conjugated secondary antibodies. To analyze Gal-7 binding to MDSCs, cells were differentiated from BM progenitors (as described below), activated with LPS (1 μg/ml; Invitrogen) for 16 h at 37 °C and incubated with rGal-7-PerCP/Cy5.5 (40 μg/ml), FITC-conjugated anti-Ly6C (green) and PE-conjugated anti-Ly6G (red) antibodies for 30 min. Cells were fixed with 4% paraformaldehyde/PBS for 15 min. Slides and cells were analyzed in an Olympus laser confocal microscope (Fluoview FV10i). At least 7 fields were analyzed for cell number and signal intensity.

### Differentiation of BM-derived MDSCs

BM-derived progenitor cells were obtained from femurs of female C57BL/6 WT, *C2gnt1*^*−/−*^ or *Mgat5*^*−/−*^ mice (6–8 weeks-old). Cells were cultured in RPMI 1640 medium supplemented with 10% FBS, 1 mM HEPES (Life Technologies), 10 ng/ml granulocyte macrophage-colony stimulating factor (GM-CSF) (R&D Systems) and antibiotics/antimycotics for 4 days. After in vitro differentiation, cells were incubated with LPS (1 μg/ml; Invitrogen) for 16 h at 37 °C in the presence or absence of rGal-7 (20 μg/ml) or lactose (30 mM; Sigma). The phenotype of MDSCs was determined by flow cytometry as described [45]. MDSCs (2 × 10^4^ cells) were co-cultured with C57BL/6 splenocytes (2 × 10^5^ cells) in RPMI 1640 supplemented with 10% FBS, 50 μM 2-ME, 1 mM HEPES, anti-CD3 mAb (1 μg/ml), anti-CD28 mAb (1 μg/ml) and antibiotics/antimycotics for 4 days. The ability of MDSCs to control T-cell proliferation was assessed by flow cytometry following incubation with 1 μM 5(6)-carboxyfluoresceindiacetate N-succinimidyl ester (CFSE; eBioscience).

### Cytokine assays

Mouse IL-12p70, IL-10, IL-1β and TGF-β_1_ ELISA sets (BD Biosciences) and mouse Gal-7 and IL-27p28 ELISA kits (R&D Systems) were used following manufacturer’s instructions.

### Migration assays

M-MDSCs (1 × 10^5^) were plated in 250 µl of DMEM on top of the filter membrane (8 µm) in a transwell insert, and 500 µl of conditioned media from Kera-308 previously treated or not with hepatocyte growth factor (HGF) alone or with anti-CXCL1 blocking antibody (see Supplementary Materials and Methods) were added into the bottom of the lower chamber in a 24-well plate. MDSCs were incubated for 16 h at 37 °C and 5% CO_2_ to allow cells to partially migrate towards the lower chamber. To quantify the number of migrated MDSCs in the lower layer of the transwell, the insert was removed from the plate and media. Then, remaining cells that have not migrated were removed from the top of the membrane using a cotton-tipped applicator. Transwells were incubated at room temperature for 2–5 min in a solution of Cristal Violet 0.5% and Methanol 20% in water (for staining and fixation). After removing crystal violet excess, transwell membranes were allowed to dry for 24 h. An inverted microscope was used to count cells (5 fields per membrane). Average number of cells that have migrated through the membrane and remain attached on the underside of the membrane was recorded. In addition, number of cells that have passed through the membrane to the lower chamber media were counted with a Neubauer Chamber and a Cell coulter. Cell migration analysis was performed under blind conditions. The estimated total number of migrated cells was calculated as follows: N of migrated M-MDSCs (transwell) = (Mean cell count in each field/0.002 cm^2^) * 0.33 cm^2^.

### Data mining analysis of *LGALS7* gene in human neoplastic diseases

For a comparative analysis of *LGALS7* gene expression and copy number variation in different human cancers, data from 9,186 primary tumors and their associated normal adjacent tissue samples obtained from The Cancer Genome Atlas (TCGA) Pan-Cancer Analysis Project were analyzed. Clinical and pre-processed data (RNA-Seq RSEM normalized counts, CNV profiles) were retrieved from the UCSC Xena Browser (http://xena.ucsc.edu/). Data were integrated and visualized using the R software. A panel of NMSC lesions and normal skin samples (E-MTAB-5678; [46]) was also analyzed using the EdgeR R/Bioconductor package for data pre-processing of the RNA-Seq transcriptome provided as the number of counts mapped to each gene. Average *LGALS7* and *LGALS7B* mRNA expression profiles (log2 count per millions) were employed for further statistical analysis with R software.

### Skin tissue from NMSC patients and healthy donors

Formalin-fixed paraffin-embedded surgical skin specimens from NMSC patients and healthy donors were obtained from the Dermatology Department at Hospital Italiano (Buenos Aires, Argentina). Samples were obtained following patients’ informed consent, and protocols were approved by the Ethics Committee of Hospital Italiano and IBYME.

### Statistical analysis

Prism software (GraphPad) was used for statistical analysis. Two groups were compared with the Student ´s *t* test for paired and unpaired data. Two-way ANOVA and Dunnett’s or Tukey post-tests were used for multiple comparisons. Nonparametric analysis was performed using Mann–Whitney *U*-test. *P* values of 0.05 or less were considered significant.

### Reporting summary

Further information on research design is available in the Nature Portfolio Reporting Summary linked to this article.

## Results

### Genotoxic and inflammatory insults trigger Gal-7 expression

**Fig. 1:**
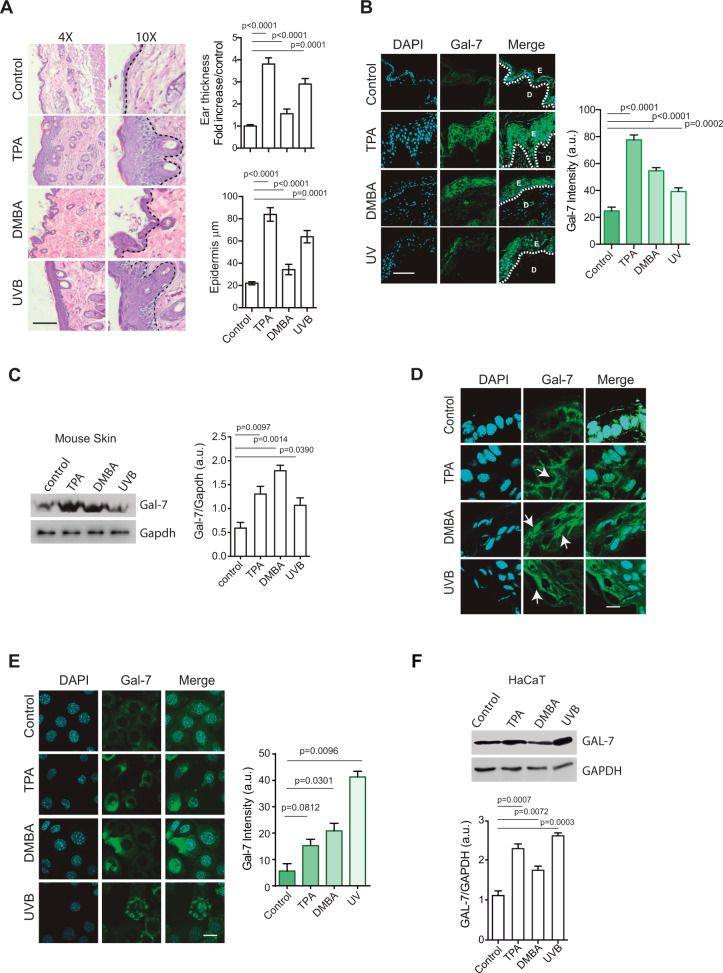
Gal-7 expression is increased in response to genotoxic and inflammatory insults. **A**–**D** Ears from WT mice were treated with TPA (20 nmol) or DMBA (200 nmol), exposed to UV radiation (average of 3.1 KJ/m^2^ UVB), or remained untreated (control). **A** Evaluation of skin damage and inflammation. Representative images of *H&E* staining (left panel; bar represents 1000 µm) and quantification of ears and epidermis thickness (right panels; mean ± SEM; 3 independent experiments) are shown. **B** Gal-7 expression and localization assessed by immunofluorescence staining. Representative images (left panel; bar represents 100 µm) and quantification (right panel; mean ± SEM; 3 independent experiments) are shown. **C** Gal-7 expression evaluated by Western blot in total protein extracts (30 µg of protein) from mice ears. Representative Western blot (left panel) and quantification of band intensity (right panel; mean + SEM; 3 independent experiments) are shown. **D** Gal-7 expression and localization assessed by immunofluorescence staining. Representative images are shown (bar represents 10 µm; arrows show Gal-7 nuclear staining). **E**, **F** HaCaT cell line was treated with TPA (20 nmol), DMBA (200 nmol), UV light (average dose of 3.1 KJ/m2 UVB) or remained untreated (control). **E** Gal-7 expression evaluated by immunofluorescence staining. Representative images (left panel; bar represents 10 μm) and quantification (right panel; mean ± SEM; 3 independent experiments) are shown. **F** Gal-7 expression evaluated by Western blot in total protein extracts (30 µg of protein). Representative Western blot assays (left panel) and determination of band intensities (right panel; mean + SEM; 4 independent experiments) are shown.

Given the role of Gal-7 in skin homeostasis and inflammation [16–20], we first studied its regulated expression in response to different insults. scRNA-seq data analysis from Mouse Cell Atlas showed preferential expression of *LGALS7* mRNA in keratinocytes (KCs; Supplementary Fig. S1A). Exposure to UVB radiation or topical administration of TPA or DMBA resulted in pronounced inflammation and increased epidermis thickness in mouse skin (Fig. 1A), as well as increased Gal-7 protein expression compared to non-treated mice (Fig. 1B, C). Gal-7 was localized both in the intracellular and extracellular compartments in treated mouse skin (primarily KCs) (Fig. 1D). Gal-7 expression also increased in human immortalized KCs (HaCaT) upon exposure to radiation or chemical stimuli (Fig. 1E, F), highlighting the importance of these factors in regulating Gal-7 expression in KCs.

### Heightened Gal-7 expression accelerates and aggravates skin carcinogenesis

**Fig. 2:**
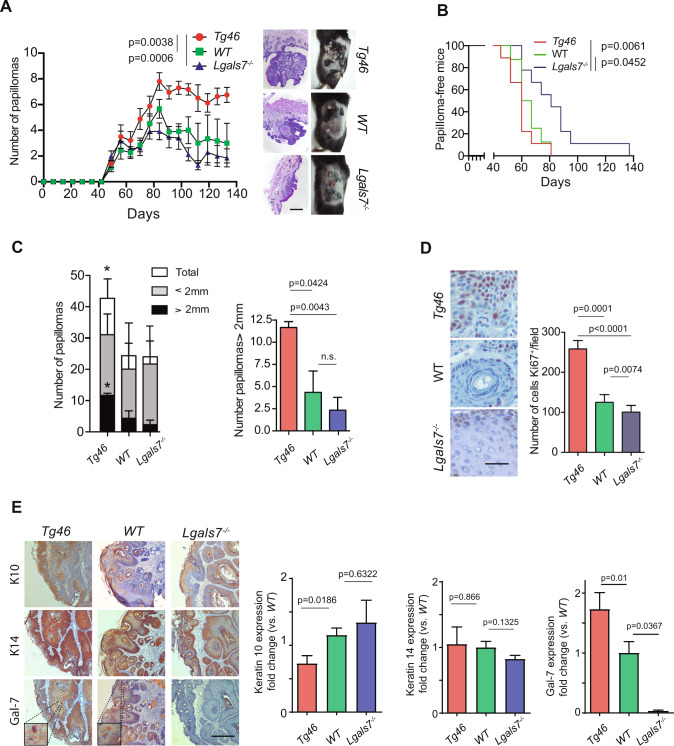
Gal-7 favors tumor growth in a murine skin carcinogenesis model. *Lgals7*^*−/−*^, WT and *Tg46* mice were subjected to a two-stage carcinogenesis protocol (DMBA/TPA). **A** Weekly monitoring of tumor growth. Tumor number in each experimental group (left panel; mean ± SEM of 4 independent experiments, each experiment with *n* = 5 mice per group) and representative images of H&E staining of papillomas (bar represents 1000 µm) and mice shaved backs (right panel) are shown. **B** Percentage of papilloma-free mice during the course of the experiment (representative of 4 independent experiments). **C** Number of tumors in each experimental group classified according to lesion size (total, <2 mm and >2 mm; mean ± SEM; 3 independent experiments) at the end of carcinogenesis protocol. Right panel, results corresponding to papillomas>2 mm are detailed. *p < 0.05. **D** Ki67 expression in papillomas from *Lgals7*^*−/−*^, WT and *Tg46* mice at the end point of carcinogenesis protocol, evaluated by immunohistochemistry. Representative images (left panel; bar represents 100 µm) and quantification of Ki67^+^ cells (right panel; mean ± SEM; 3 independent experiments) are shown. **E** Expression of K10, K14 and Gal-7 in papillomas from *Tg46*, WT and *Lgals7*^*−*/*−*^ mice evaluated by immunohistochemistry. Representative images of 10 samples per group (left panel; bar represents 1 mm) and quantification (right panels; mean ± SEM; 3 independent experiments) are shown. In panel **A**, *p* values correspond to the end point of the experiment.

Given the sensitivity of Gal-7 to skin insults, we investigated its contribution to skin carcinogenesis using a two-stage skin carcinogenesis model. Tumor initiator DMBA and tumor promoter TPA were administered topically and sequentially in WT and *Lgals7*^*−/−*^ mice, and in *Tg46* mice, which constitutively express Gal-7 under the K14 promoter (Supplementary Fig. S1B). Lack of Gal-7 or its overexpression was confirmed by Western blot, ELISA and immunohistochemistry (Supplementary Fig. S1C–E). Interestingly, constitutive expression of Gal-7 in KCs (*Tg46* mice) resulted in a higher number of papillomas per mouse (Fig. 2A) and an earlier appearance of tumors (Fig. 2B), compared to WT and *Lgals7*^*−/−*^animals. At day 60, almost 80% of *Tg46* animals developed at least one tumor, compared to 50% of WT and 25% of *Lgals7*^*−/−*^ mice (Fig. 2B), with a considerably higher number of tumors per animal from day 80 onwards. On the contrary, *Lgals7*^*−/−*^ mice showed delayed occurrence of skin lesions compared to WT and *Tg46* animals (Fig. 2B), and consistently low or even decreasing number of papillomas per mouse (Fig. 2A). Considerable differences were found between *Tg46* and WT or *Lgals7*^*−/−*^ animals in the total number of papillomas and particularly in lesions larger than 2 mm (Fig. 2C). A substantial increase in the number of Ki67^+^ cells, indicative of proliferative activity, was detected in papillomas from *Tg46* compared to WT or *Lgals7*^*−/−*^ mice at the end of the carcinogenesis process (Fig. 2D), which was accompanied by altered expression of K10 (a suprabasal restricted keratin), but not of the basal-restricted K14 (Fig. 2E). At that point, Gal-7 expression remained higher in *Tg46* lesions compared to WT tumors both at the protein and mRNA levels (Fig. 2E and Supplementary Fig. S1F).

### Gal-7 expression reprograms the immune landscape of non-melanoma skin tumors

**Fig. 3:**
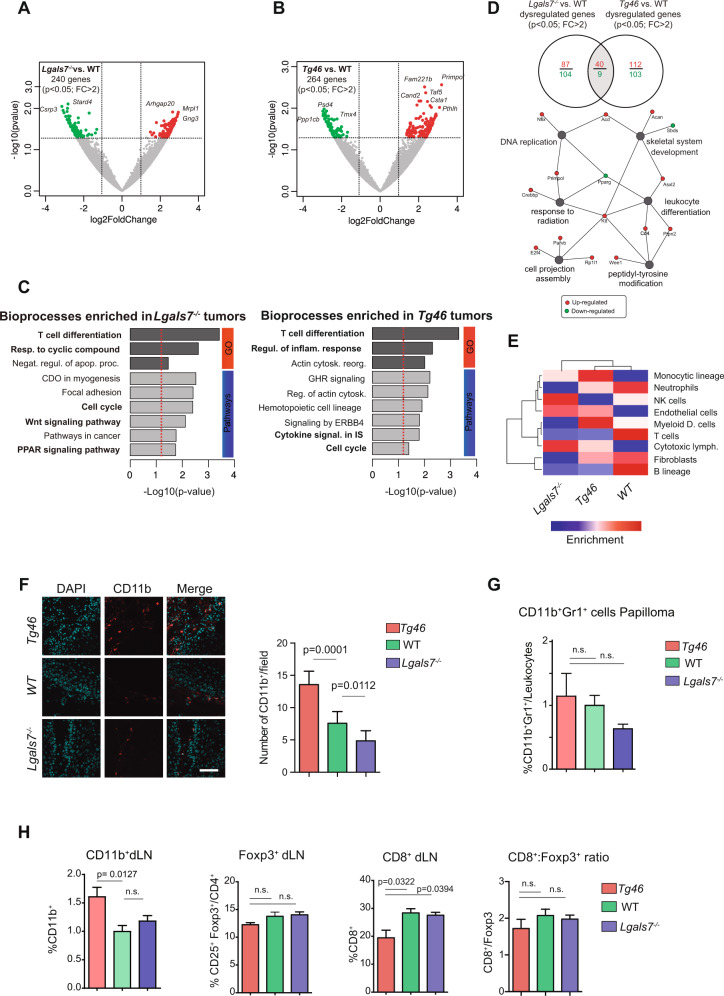
Enhanced Gal-7 expression shapes the immune landscape in murine skin papillomas. Volcano-plots from analysis of RNAseq data from *Lgals7*^*−*/*−*^, *Tg46* and WT papillomas, showing 240 and 264 genes differentially expressed in *Lgals7*^*−/−*^ (**A**) and *Tg46* (**B**) lesions, respectively, compared with WT tumors (*p* < 0.05, FC > 2). **C** Functional enrichment analysis of RNAseq data. Bioprocesses found enriched in *Lgals7*^*−*/*−*^ (left panel) and *Tg46* (right panel) tumors, compared with their WT counterparts, are shown. **D** Genes found to be dysregulated in *Lgals7*^*−/−*^ or *Tg46* lesions compared with WT tumors, and network-based pathway enrichment analysis. **E** Heat map analysis of tumor-infiltrating immune cells. **F** Analysis of CD11b^+^ cells in papillomas from *Tg46*, WT and *Lgals7*^*−/−*^ mice, evaluated by immunofluorescence. Representative images (left panel; CD11b^+^ red, DAPI blue; bar represents 100 µm) and determination of CD11b^+^ cells (right panel; mean ± SEM; 3 independent experiments) are shown. **G** Percentage of CD11b^+^Gr1^+^ cells in papillomas from *Tg46*, WT and *Lgals7*^*−/−*^ mice, evaluated by flow cytometry (mean ± SEM; 3 independent experiments). **H** Immune cell populations in tumor draining lymph nodes (dLN) from *Tg46*, WT and *Lgals7*^*−/−*^ mice at the end point of carcinogenesis protocol, analyzed by flow cytometry. Percentage of CD11b^+^, CD4^+^ CD25^+^ Foxp3^+^ and CD8^+^cells^,^ and CD8^+^/ CD4^+^ CD25^+^ Foxp3^+^ ratio (mean ± SEM; 4 independent experiments) are shown.

To gain insights into the mechanisms underlying Gal-7-induced skin carcinogenesis, we next performed RNA-seq analysis in WT, *Lgals7*^*−/−*^ and *Tg46* mice skin papillomas, which revealed clusters of 240 and 264 genes differentially expressed in distinct mouse strains (Fig. 3A, B, and Supplementary RNAseq Datasheet 1). Despite isolated changes, no substantial alterations were observed in the expression of most skin-specific genes (Supplementary Fig. S1G). Noteworthy, the suprabasal-restricted proteins K1 and Repetin were identified as differentially expressed in papillomas from *Tg46* animals compared to WT mice (*p* = 0.029 and *p* = 0.008, respectively), while the expression of basal-restricted K14 remained unaltered. No differences were found in the expression of other genes associated with KC differentiation (Supplementary Fig. S1G). Of note, gene modules related to T-cell differentiation (*p* = 0.005), regulation of inflammatory response (*p* = 0.005), cytokine signaling (*p* = 0.02), and cell cycle progression (*p* = 0.04) were enriched in *Tg46* papillomas (Fig. 3C).

Given the central roles of galectins in reprogramming antitumor immunity [47] and the association of Gal-7 with an immune-related transcriptional profile (Fig. 3C), we then studied the impact of this lectin in shaping the immune landscape of skin tumors. RNAseq data showed a higher representation of cells of the myelo-monocytic lineage in tumors from *Tg46* mice, compared to lesions from *Lgals7*^*−/−*^ animals which were enriched in NK cells and cytotoxic T cells (Fig. 3E). Consistently, papillomas from *Tg46* mice showed increased frequency of tumor-infiltrating CD11b^+^ cells (Fig. 3F). Flow cytometry analysis of tumor-infiltrating leukocytes showed increased percentage of cells expressing both CD11b and Gr-1 markers (CD11b^+^Gr1^+^cells), characteristic of myeloid-derived suppressor cells (MDSC) in *Tg46* tumors (Fig. 3G). Accordingly, analysis of draining lymph nodes (dLNs) revealed a significantly higher percentage of CD11b^+^ cells in *Tg46* mice compared with those from WT or *Lgals7*^*−/−*^ mice (Fig. 3H). Despite a substantial drop in the percentage of effector CD8^+^ T cells in *Tg46* versus WT or *Lgals7*^*−/−*^ mice, no significant differences were observed in the percentage of CD4^+^CD25^+^Foxp3^+^ T regulatory (Treg) cells or in the CD8^+^/CD4^+^CD25^+^Foxp3^+^ ratio (which is indicative of the relationship of effector and Treg cell responses) among groups (Fig. 3H), highlighting the importance of Gal-7 in influencing the myeloid versus lymphoid T-cell compartments.

**Fig. 4:**
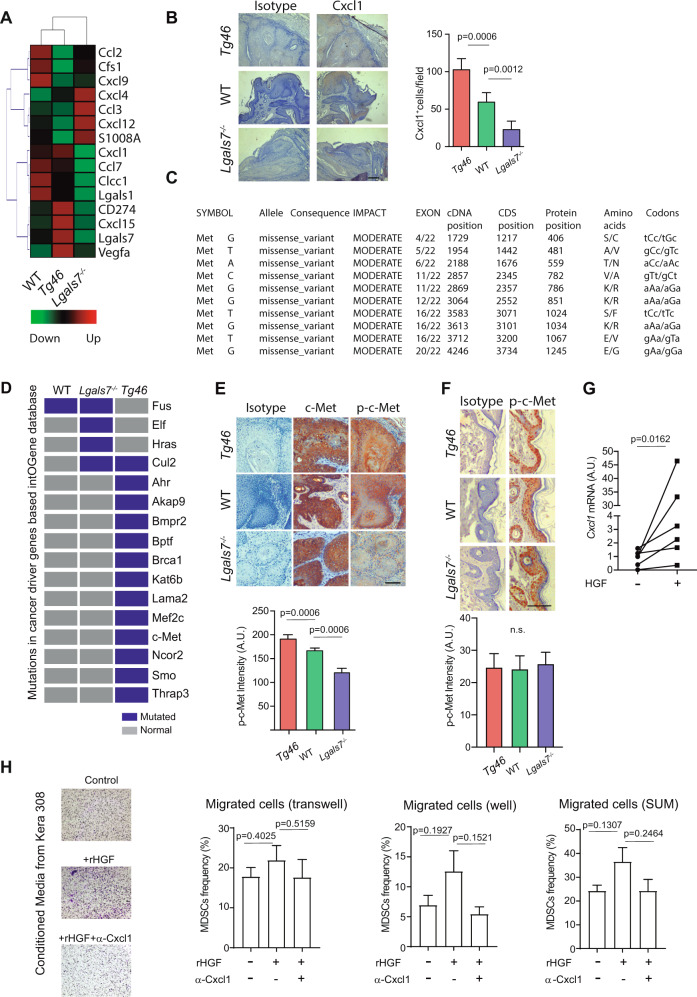
c-Met-dependent Cxcl1 expression contributes to Gal-7-driven recruitment of immunoregulatory myeloid cells to the TME. **A** Heatmap analysis of chemokine and cytokine genes expression levels in papillomas from WT, *Tg46* and *Lgals7*^*−*/*−*^ animals, obtained from RNAseq data. **B** Cxcl1 expression level evaluated by immunohistochemistry, in papillomas from *Tg46*, WT and *Lgals7*^*−/−*^mice. Representative images (upper panel; bar represents 100 µm) and determination of Cxcl1^+^ cells (lower panel; mean ± SEM; 3 independent experiments) are shown. **C**, **D** Exome-seq analysis and mutational profiles of papillomas (*n* = 3 pooled tumors per group) from *Lgals7*^*−/−*^, WT and *Tg46* mice subjected to carcinogenesis protocol. **C** Identification of different DNA missense mutations in the *c-Met* gene. **D** Missense mutations in cancer driver genes identified in each tumor type (Blue squares). Analysis of c-Met activation determined by p-c-Met and c-Met immunohistochemistry in papillomas (**E**) and normal skin (**F**) from *Tg46*, WT and *Lgals7*^*−/−*^ mice. Representative imagines (upper panels; bars represent 100 µm) and quantification (lower panels; mean ± SEM; 3 independent experiments) are shown. **G** *Cxcl1* mRNA evaluated by real time qPCR, in Kera-308 cells treated or not with 10 ng/ml of HGF. Results are presented in arbitrary units (AU) relative to Gapdh mRNA (mean ± SEM; 3 independent experiments). **H** Migration assays of BM-derived MDSC in the presence of conditioned media from Kera-308 cells that had been incubated with or without HGF (10 ng/ml) for 16 h. The impact of Cxcl1 on the migration of MDSC was evaluated by adding anti-Cxcl1 blocking antibody to migration media (mean ± SEM; 3 independent experiments).

Seeking for possible mechanisms underlying the increased myeloid cell representation in the tumor microenvironment (TME) and tumor-dLN of *Tg46* mice, we examined RNAseq data, which revealed increased transcription of chemokine and cytokine genes implicated in myeloid cell recruitment in lesions from *Tg46* compared to those from WT mice (i.e., *Cxcl1* and *VEGFa*), and in papillomas from WT mice compared with their *KO* counterpart (i.e., *Cxcl1*, *Ccl7* and *Clcc1;* Fig. 4A). Particularly interesting, *Cxcl1* expression pattern followed that of Gal-7. Accordingly, tumors overexpressing Gal-7 (*Tg46*) showed a considerably higher percentage of Cxcl1^+^ cells than WT papillomas, and even higher than *Lgals7*^*−/−*^ tumors (Fig. 4B).

Through recruitment of CD11b^+^Gr1^+^ myeloid cells to the TME, Cxcl1 contributes to cancer survival and metastasis [48, 49]. Notably, Cxcl1 secretion may be controlled by activation of HGF receptor c-Met [49, 50], which aberrant signaling has been associated with cancer progression and invasion [51, 52]. Notably, exome-seq analysis of DNA from WT, *Lgals7*^*−/−*^ and *Tg46* mouse papillomas (Supplementary Dataset 2 Mutational Profiles) showed high variability of genomic missense mutations particularly localized in the c-Met gene in lesions from *Tg46* mice (Fig. 4C). Indeed, *Tg46* lesions presented the highest number of non-synonymous mutations on target genes described either as cancer-driver genes or as potential drivers, as defined by the IntOGene database (Fig. 4D and Supplementary Fig. S2A). Mutations in *Tg46* tumors impinged on cancer driver genes mostly related to phosphoinositide 3-kinases (PI3K)-Akt signaling (e.g., *c-Met*) and DNA repair (e.g., *Brca1*) pathways (Fig. 4C, D and Supplementary Fig. S2B). Moreover, *Tg46* papillomas showed an increased number of single base substitutions (Supplementary Fig. S2C) and a unique mutational profile characterized by a greater number of A > G:T> C transitions (*p* < 0.05), and lower number of A > C:T > G transversions (*p* < 0.05), compared with WT lesions (Supplementary Fig. S2D). Seeking for possible mechanisms that could explain genomic instability, the DNA repair protein PCNA was identified as a possible Gal-7 interacting partner in pull-down experiments followed by mass spectrometry analysis (Supplementary Fig. S2E). Co-immunoprecipitation (Fig. S2F) and immunofluorescence staining (Supplementary Fig. S2G) further supported this finding. Nevertheless, in in vitro functional assays, neither nucleotide excision repair (Supplementary Fig. S2H) nor mismatch repair (Supplementary Fig. S2I), which are PCNA-mediated mechanisms, were affected by augmented Gal-7 levels. Thus, alternative mechanisms might potentially be affected by Gal-7/PCNA interaction.

Given the association between c-Met pathway activation and Cxcl1-mediated recruitment of myeloid cells to the TME [49], the identification of mutated *c-Met* as a prominent cancer driver gene in *Tg46* mouse papillomas (Fig. 4C, D) and the higher percentage of Cxcl1^+^ cells in these tumors (Fig. 4B), we then explored if abnormal c-Met activation could be responsible for the increased expression of Cxcl1. We found increased phosphorylation of c-Met in tumors (Fig. 4E), but not in normal skin (Fig. 4F) of *Tg46* versus WT mice, and in WT versus *Lgals7*^*−/−*^mice. Notably, in the presence of HGF (c-Met ligand), KCs showed a significant increase in *Cxcl1* expression (Fig. 4G), suggesting that c-Met activation may indeed control *Cxcl1* expression in KCs. We then evaluated if increased Cxcl1 levels could be responsible, at least in part, for the greater number of infiltrating CD11b^+^ cells in migration assays. Although not statistically significant, supernatants from HGF-treated KCs increased migration of BM-derived MDSCs, compared to supernatants from untreated KCs; interestingly antibody-mediated Cxcl1 blockade attenuated this effect (Fig. 4H). Thus, c-Met activation driven-Cxcl1 expression might contribute, at least in part, to Gal-7-driven recruitment of myeloid cells to the TME.

### Gal-7 impacts on the differentiation and immunosuppressive activity of tumor-infiltrating myeloid cells

**Fig. 5:**
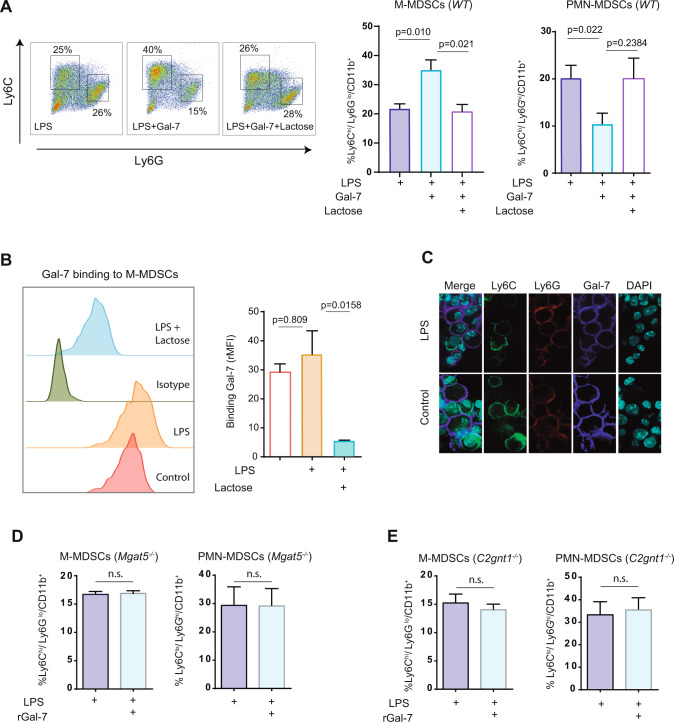
Gal-7 binding to myeloid cells promotes polarization toward a monocytic profile. **A** WT BM-derived MDSCs were activated with LPS in the presence (LPS + Gal-7) or absence (LPS) of rGal-7 (20 µg/ml) and with or without lactose (30 mM). Representative dot plots (left panel) and quantification (right panels) of the frequencies of MDSCs subpopulations obtained, determined according to Ly6C and Ly6G markers in CD11b^+^ gated cells (mean ± SEM of 3 independent experiments), are shown. **B** Binding of Gal-7 to WT BM-derived MDSCs activated or not with LPS, in the presence or absence of lactose, evaluated by flow cytometry. Left panel, representative histogram. Right panel, quantification (mean ± SEM; 4 independent experiments). **C** Gal-7 binding to non-activated (control) and activated (LPS) BM-derived MDSCs, evaluated by immunofluorescence staining (Gal-7, blue; Ly6C, green; Ly6G, red). Representative images of 3 independent experiments are shown (bar represents 10 µm). Frequencies of MDSCs subpopulations obtained when BM-derived MDSCs from *Mgat5*^*−/−*^ (**D**) and from *C2gnt1*^*−/−*^ (**E**) mice were activated with LPS in the presence or absence of rGal-7 (20 µg/ml) (mean ± SEM of 4 independent experiments).

Considering the prominent expression of Gal-7 in the extracellular skin microenvironment (Fig. 1D) and the higher frequency of CD11b^+^Gr1^+^ cells in tumors and tumor dLNs of *Tg46* mice (Fig. 3G, H), we then investigated the impact of this lectin in the biology of these cells. Activation of BM-derived CD11b^+^ cells in the presence of rGal-7 led to a considerable increase in CD11b^+^Ly6C^hi^Ly6G^lo^ (monocytic MDSCs; M-MDSCs) and a reduction in CD11b^+^Ly6C^lo^Ly6G^hi^ cells (polymorphonuclear MDSCs; PMN-MDSCs) (Fig. 5A). Flow cytometry and confocal microscopy experiments confirmed association of Gal-7 to the surface of activated MDSCs (Fig. 5B, C). Gal-7 binding to MDSCs and further polarization toward a monocytic profile were both prevented by lactose, a galectin-specific disaccharide, suggesting the involvement of protein-glycan interactions in these effects (Fig. 5D, E). Accordingly, when we used BM progenitors from mice lacking Mgat5 (*Mgat5*^*−/−*^), an enzyme required for complex N-glycan branching, or from mice lacking C2gnT1 (*C2gnt1*^*−/−*^), a glycosyltransferase essential for core 2-O-glycan elongation, Gal-7 effects were prevented, suggesting the contribution of specific glyco-epitopes on Gal-7 function (Fig. 5F). Thus, Gal-7-glycan interactions may tilt MDSC polarization toward a monocytic (CD11b^+^Ly6C^hi^Ly6G^lo^) profile.

**Fig. 6:**
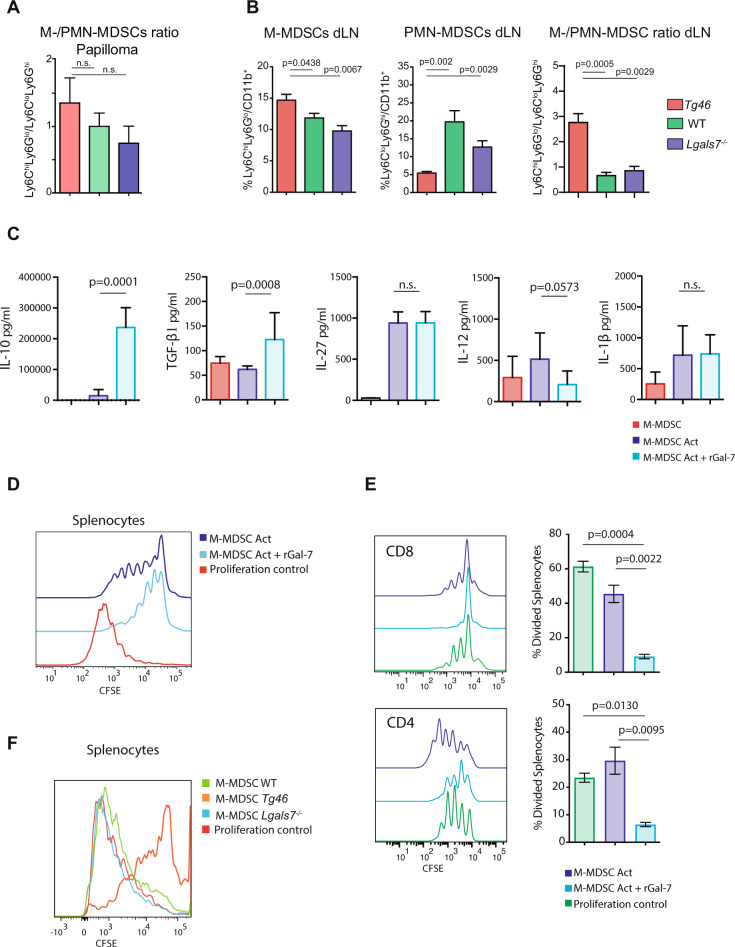
Gal-7 influences polarization and immunosuppressive function of myeloid cells. **A**, **B** Impact of Gal-7 on MDSC polarization in the TME. **A** M-MDSC/PMN-MDSC ratio in papillomas from *Tg46*, WT and *Lgals7*^*−/−*^ mice, evaluated by flow cytometry (mean ± SEM; 3 independent experiments). **B** Percentage of M-MDSCs and PMN-MDSCs, and M-MDSC/PMN-MDSC ratio in tumor draining lymph nodes (dLN) from *Tg46*, WT and *Lgals7*^*−/−*^ mice at the end point of carcinogenesis protocol, analyzed by flow cytometry (mean ± SEM; 4 independent experiments). **C**–**F** Impact of Gal-7 on MDSC immunosuppressive function. **C**–**E** BM-derived MDSCs were activated in the presence or absence of rGal-7. **C** Determination of secreted cytokines (IL-10, TGF-β_1_, IL-27, IL-12 and IL-1β) by ELISA (mean ± sem; 5 independent experiments). Analysis of the immune inhibitory activity of BM-derived M-MDSCs (sorted) over (**D**) splenocytes or (**E**) CD4^+^ and CD8^+^ T cells, evaluated in lymphocyte proliferation assays (mean ± SEM; 3 independent experiments). **F** T-cell inhibitory activity of M-MDSCs (2 ×104 cells) isolated by sorting from spleen of *Tg46*, WT and *Lgals7*^*−/−*^ animals at the endpoint of the carcinogenesis protocol, over WT splenocytes (2 × 105 cells), as determined by T-cell proliferation assays (representative of 3 experiments).

Next, we characterized tumor-infiltrating leukocytes from mice subjected to the carcinogenesis protocol by flow cytometry. Although not statistically significant, results showed a trend toward a higher, M-MDSC/PMN-MDSC ratio in tumors overexpressing Gal-7 (Fig. 6A). Accordingly, analysis of tumor dLNs revealed a significantly higher percentage of M-MDSC, reduced frequency of PMN-MDSC and higher M-MDSC/PMN-MDSC ratio in tumor-bearing *Tg46* mice compared with WT or *Lgals7*^*−/−*^ mice (Fig. 6B). Thus, Gal-7 reprograms the TME not only by enhancing recruitment but also by modulating differentiation of M-MDSC.

To investigate the impact of Gal-7 in the immunosuppressive activity of M-MDSCs, these cells were exposed or not to rGal-7 during activation. Results revealed a selective increase in the secretion of immunosuppressive (IL-10, TGF-β_1_), but not pro-inflammatory (IL-12, IL-1β) cytokines (Fig. 6C). Moreover, BM-derived M-MDSCs exposed to rGal-7 showed increased immune inhibitory activity when added to splenocytes (Fig. 6D) or to purified CD4^+^ or CD8^+^ T cells (Fig. 6E). Furthermore, splenic M-MDSCs isolated from tumor-bearing *Tg46* mice showed increased T-cell inhibitory activity, as compared with those from WT or *Lgals7*^*−/−*^ mice (Fig. 6F). Thus, Gal-7-driven programs may tilt myeloid cell polarization toward a monocytic profile and accentuate their immunoregulatory function.

### Myeloid cells are critical mediators of Gal-7-driven skin carcinogenesis

**Fig. 7:**
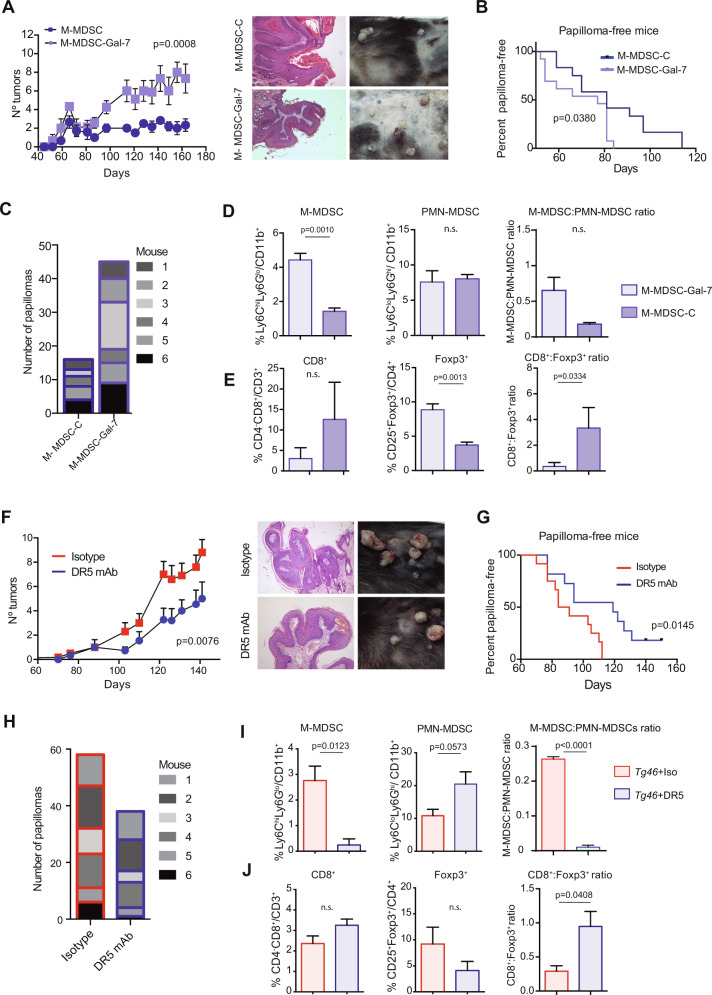
Gal-7-drives skin carcinogenesis by targeting myeloid cells. **A**–**E** M-MDSCs differentiated from WT BM cells and activated in the presence (M-MDSC-Gal-7) or absence (M-MDSC-C) of rGal-7 were injected weekly in *Lgals7*^*−/−*^ mice, during the two-stage carcinogenesis protocol. **A** Tumor growth was weekly monitored and scored. Average tumor number in each experimental group (left panel; mean ± SEM; 3 independent experiments) and representative images of mouse shaved backs and H&E of papillomas (right panel) are shown. **B** Frequency of papilloma-free mice. **C** Total tumor number in each experimental group (*n* = 6 animals per group, from one representative experiment are shown). **D**, **E** Characterization of tumor infiltrate in papillomas from *Lgals7*^*−/−*^ animals injected with M-MDSC-C or M-MDSC-Gal-7. Determination of M-MDSCs and PMN-MDSCs frequencies and of M-/PMN-MDSC ratio (**D**), and assessment of CD8^+^ and CD4^+^CD25^+^Foxp3^+^ cell frequencies, as well as CD8^+^/CD4^+^CD25^+^ Foxp3^+^ ratio (**E**) are shown (mean ± SEM; 3 independent experiments). **F**–**J** *Tg46* mice were treated weekly with anti-DR5 mAb or with isotype control mAb beginning from the first month of the carcinogenesis protocol (*n* = 3, 5 and 6 animals per group; 3 independent experiments). **F** Tumor growth was weekly monitored and scored. Curves corresponding to tumor progression of individual animals (left panels) and representative images of one animal from each experimental group and H&E of papillomas (right panel) are shown. **G** Frequency of papilloma-free mice is shown. **H** Total tumor number in each experimental group (*n* = 6 animals per group, from one representative experiment are shown). **I**, **J** Characterization of tumor infiltrate in papillomas from *Tg46* animals treated with anti-DR5 mAb or with isotype control. Frequencies of M-MDSCs and PMN-MDSCs, and M-/PMN-MDSC ratio (**I**) and determination of the frequencies of CD8^+^ and CD4^+^ CD25^+^ Foxp3^+^ cells, and CD8^+^/ CD4^+^ CD25^+^ Foxp3^+^ ratio (**J**) are shown (mean ± SEM; 3 independent experiments). In panels **A** and **F** *p* values correspond to the end point of the experiment.

To investigate the relevance of Gal-7 in accelerating skin carcinogenesis through modulation of M-MDSCs activity in vivo, we activated these cells in the presence (M-MDSCs-Gal-7) or absence (M-MDSCs-C) of rGal-7 and inoculated them weekly into *Lgals7*^*−/−*^ mice during the two-stage carcinogenesis protocol (Supplementary Fig. S3A). DMBA/TPA-treated *Lgals7*^*−/−*^ animals adoptively transferred with M-MDSC-Gal-7 developed a considerably higher number of papillomas compared with *Lgals7*^*−/−*^ mice receiving M-MDSC-C (Fig. 7A) and displayed a similar phenotype to that observed in DMBA/TPA-treated WT mice (Supplementary Fig. S3B). Notably, *Lgals7*^*−/−*^ mice receiving M-MDSC-Gal-7, showed not only an earlier appearance of papillomas (Fig. 7B), but also an increased number of total papillomas, compared to those receiving M-MDSCs-C (Fig. 7C). Papillomas developed in *Lgals7*^*−/−*^ mice which received M-MDSC-Gal-7 showed higher number of Ki67^+^ proliferating cells compared to tumors from mice injected with M-MDSC-C (Supplementary Fig. S3C). However, we detected a comparable intensity of K10 and K14 staining in different animal groups (Supplementary Fig. S3D). Analysis of tumor infiltrating cells revealed a higher M-MDSC frequency (% of Ly6C^hi^Ly6G^lo^ cells from infiltrating CD11b^+^ cells; Fig. 7D and Supplementary Fig. S3E) and a greater percentage of Tregs (CD25^+^Foxp3/CD4^+^cells), which led to a lower CD8^+^/CD4^+^CD25^+^Foxp3^+^ ratio, indicative of attenuated T cell responses (Fig. 7E and Supplementary Fig. S3E) in papillomas from *Lgals7*^*−/−*^ animals receiving M-MDSC-Gal-7 compared with those receiving M-MDSC-C. Thus, Gal-7-educated M-MDSCs foster local immunosuppression and exacerbate skin carcinogenesis in vivo.

To further investigate the role of MDSCs as critical mediators of Gal-7-driven skin carcinogenesis, we depleted these cells in DMBA/TPA-treated *Tg46* mice by weekly injection of anti-DR5 mAb (which induces TRAIL-mediated apoptosis of MDSCs) (Supplementary Fig. S3F). Depletion of 70% of M-MDSCs was confirmed in the spleen of tumor-bearing *Tg46* mice (Supplementary Fig. S3G). Anti-DR5 mAb-treated *Tg46* mice showed lower number of tumors per animal (Fig. 7F), delayed appearance of papillomas (Fig. 7G) and lower number of total tumors (Fig. 7H), compared to those treated with control mAb. Moreover, the number of Ki67^+^ cells, indicative of proliferative index, dropped significantly in lesions from anti-DR5 mAb-treated *Tg46* mice (Supplementary Fig. S3H). However, we found comparable K10 and K14 expression in lesions from *Tg46* mice regardless of mAb treatment (Supplementary Fig. S3I). Papillomas from anti-DR5 mAb-treated *Tg46* mice showed reduced frequency of M-MDSCs and increased proportion of PMN-MDSCs, leading to a significant drop in the M-MDSC/PMN-MDSC ratio (Fig. 7I), and higher T effector/Treg ratio (CD8^+^/CD4^+^CD25^+^Foxp3^+^ ratio), indicative of greater effector T-cell responses, compared to those treated with the control mAb (Fig. 7J). Altogether these results highlight the relevance of myeloid cells as critical targets of the immunoregulatory activity of Gal-7 during skin carcinogenesis.

### Heightened GAL-7 expression delineates malignant and pre-malignant lesions in human NMSC

**Fig. 8:**
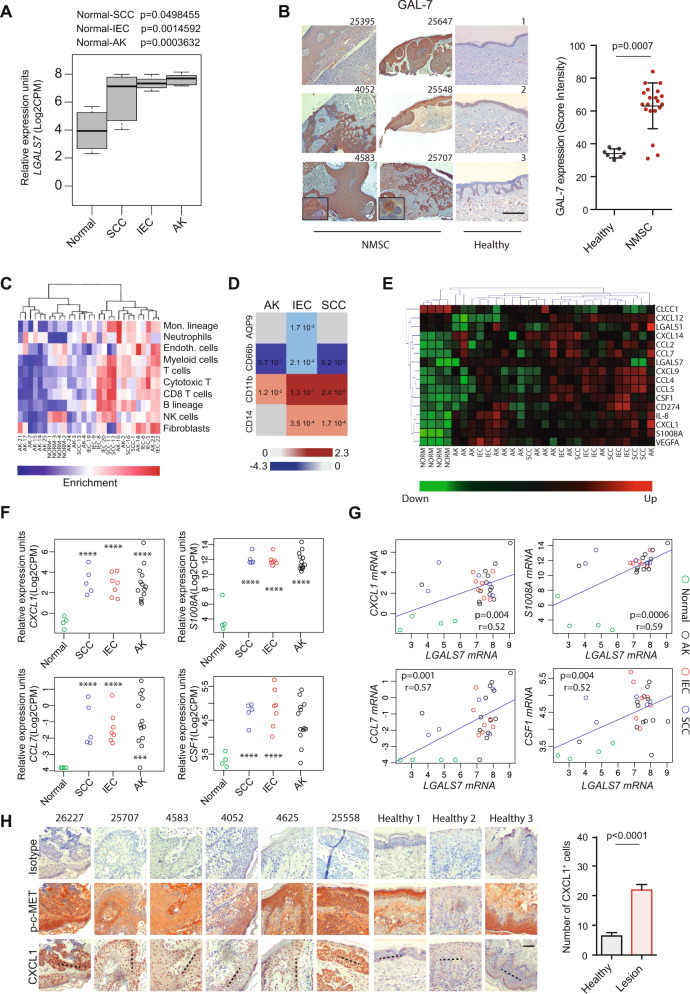
Heightened GAL-7 expression delineates human non-melanoma cutaneous malignancies. **A**, **B** GAL-7 expression in human NMSC and normal skin, evaluated in two different cohorts. **A** *LGALS7* gene expression in a panel of 4 normal skin samples and 25 NMSC lesions from E-MTAB-5678 dataset. SCC, squamous cell carcinoma (*n* = 5 patients); IEC, intraepidermal carcinoma (*n* = 7 patients); AK actinic keratosis (*n* = 13 patients). **B** GAL-7 protein expression evaluated by immunohistochemistry in skin samples from a cohort of 20 NMSC patients and 7 healthy donors. Representative images (left panel; bar represents 1 mm) and quantification of staining intensity (right panel; mean ± SEM) are shown. **C**–**E** RNAseq dataset from 4 human normal skin samples and 25 NMSC lesions (E-MTAB-5678) was analyzed. **C** Heatmap analysis of tumor-infiltrating cells in each sample. **D** Heatmap analysis of human M-MDSCs (CD11b and CD14) and PMN-MDSCs (CD66b and AQP9) marker expression levels in NMSC lesions compared to normal skin samples. **E** Heatmap analysis of chemokine and cytokine genes expression in human NMSC samples. *CXCL1*, *S100A8*, *CCL7* and *CSF1* mRNA expression levels (**F**) and correlations with *LGALS7* expression (**G**) in human NMSC analyzed from RNAseq data set (E-MTAB-5678). **H** Cxcl1 and phospho c-Met (p-c-Met) levels, determined by immunohistochemistry, in a cohort of patients diagnosed with NMSC (*N* = 20) and healthy donors (*N* = 7). Representative images (left panel; quantification across dotted line, bar represents 100 µm) and quantification of Cxcl1^+^ cells (right panel; mean ± SEM) are shown.

Given the role of Gal-7 in mouse skin carcinogenesis, and the prominent expression of this lectin in human epithelial tumors (23; Supplementary Figs. S4A and S4B), we then investigated its relevance in human NMSC. RNAseq data analysis from a cohort of NMSC patients (E-MTAB-5678 dataset) revealed increased *LGALS7* expression in neoplastic (intraepidermal carcinoma; IEC and squamous cell carcinoma; SCC) and pre-neoplastic (actinic keratosis; AK) lesions compared to normal tissue (Fig. 8A). Immunohistochemistry of tissue samples from a cohort of NMSC patients showed a substantial increase in GAL-7 protein expression in pre-malignant and malignant lesions compared with healthy skin tissue (Fig. 8B), substantiating transcriptional data. Notably, no significant variations were found in the transcriptional profile of other members of the galectin family commonly involved in tumorigenesis, between neoplastic, pre-neoplastic and healthy skin (Supplementary Fig. S4C). Thus, heightened GAL-7 expression may represent an early hallmark of carcinogenesis in human NMSC.

In line with our findings in murine carcinogenesis, NMSC malignant lesions showed enrichment in myeloid cell components as evidenced by RNAseq data analysis (E-MTAB-5678) (Fig. 8C). Indeed, IEC, SCC and AK lesions exhibited up-regulation of genes associated with monocytic myeloid cells (*CD11b* and *CD14*) and down-regulation of granulocytic markers (*CD66b* and *AQP9*) compared to healthy skin tissue (Fig. 8D). Regarding myeloid-recruiting chemokines, higher expression of *CXCL1*, *S100A8*, *CCL7* and *CSF1* was observed in lesions from AK, IEC and SCC patients compared with normal skin tissue (Fig. 8E–F). We found a strong positive correlation between *LGALS7* mRNA expression and transcription of *CXCL1*, *S1008A*, *CCL7* and *CSF1* in neoplasic skin lesions (Fig. 8G and Supplementary Fig. S4D), and higher CXCL1 expression in biopsies from NMSC patients compared to healthy skin (Fig. 8H). Interestingly, a slight increase in c-MET activation was also observed in NMSC lesions (Fig. 8H). Thus, increased GAL-7 expression in human NMSC correlates with enhanced representation of myeloid-associated programs in the TME.

## Discussion

Identification of local immunoregulatory pathways that control the interplay between malignant skin lesions and their associated microenvironment is essential for designing novel therapeutic approaches [3]. In this study we report central roles for Gal-7, an endogenous glycan-binding protein highly expressed in stratified epithelia, in skin carcinogenesis, suggesting its potential as a therapeutic target in NMSC.

Galectins have emerged as regulatory checkpoints that foster immune evasive programs in different cancer types [8]. Whereas Gal-1, Gal-3 and Gal-9 can shape myeloid cell programs by inducing tolerogenic dendritic cells (DCs) [44, 53, 54], M2-type macrophages [55] and MDSCs [56–58], the role of Gal-7 in regulating antitumor immunity has not yet been dissected. Here, we show that heightened Gal-7 expression contributes to skin carcinogenesis by fostering immunosuppressive myeloid programs. We found that this lectin induces highly immunosuppressive M-MDSCs in the TME, thus providing a possible explanation for the poor T-cell stimulatory activity of myeloid cells in SCC patients [59]. In this regard, recent evidence identified Gal-7 as a pro-metastatic gene highly expressed in immunosuppressive SCC microenvironments [60]. Additional mechanisms underlying Gal-7 effects, such as those involving altered intracellular functions should be further explored.

Lectin-glycan interactions control multiple cell processes in the TME which impact in angiogenesis, immunosuppression and metastasis [61]. Accordingly, increased frequency of core 2 O-glycans and oligomannose N-glycans has been recently reported in human NMSC lesions [62] supporting their potential role in modulating cancer cell communication with the microenvironment. Interestingly, the fact that Gal-7 exhibits 11-fold weaker affinities for LacNAc residues as compared to Gal-1 and Gal-3 [63], suggest that Gal-7 effects would prevail in pathophysiologic conditions where the concentrations of this lectin are substantially higher, leading to lower competition for the same glyco-epitopes. In this regard only Gal-7, but not Gal-1 or Gal-3, was up-regulated in human skin SCC lesions, suggesting relevant roles for this lectin in NMSC development, and highlighting a possible biomarker to delineate KC lesions from other skin tumors.

Although Gal-7 has been originally identified in KCs [12, 13], its role in KC-derived tumors has not been fully explored. Our study showed that exposure in vitro or in vivo to environmental stressors, leading to skin inflammation and carcinogenesis, resulted in substantial up-regulation of Gal-7 expression. Indeed, UVB radiation [15, 64], and pro-inflammatory cytokines [65] were reported to control Gal-7 expression in the skin. In this regard, Gal-7 was implicated in epithelial cell migration, wound re-epithelialization, and protection from skin barrier impairment [17–19, 66]. Thus, Gal-7 up-regulation may represent an adaptive stress response aimed at restoring epithelial homeostasis. However, excessive levels of this lectin as a result of sustained environmental insults may contribute to initiate a pathologic process leading to carcinogenesis.

Gain-of-function experiments comparing the tumorigenesis process in *Tg46* transgenic *versus* WT mice revealed that increased levels of Gal-7 in KCs were associated not only with earlier appearance of tumors, but also with higher number and size of lesions. On the contrary, the absence of this lectin in *Lgals7*^*−/−*^mice resulted in lower susceptibility to carcinogenesis and delayed occurrence of skin tumors compared to WT animals. Examination of possible underlying mechanisms revealed increased myeloid cell accumulation and greater M-MDSC/PMN-MDSC ratio in TME and tumor-dLN of *Tg46* mice. MDSC play crucial roles in tumor progression through mechanisms involving suppression of innate and adaptive immune responses [67]. These immature myeloid cells characterized by high heterogeneity and plasticity, inhibit T cells fitness by releasing reactive oxygen and reactive nitrogen species, by activating key enzymes, by inducing expression of co-inhibitory receptors and sustaining expansion of Tregs [68]. Induction of immature myeloid cells has been proposed to contribute as an initial step in facilitation of skin tumor formation [69]. Here, we found that Gal-7 binding to myeloid cells enhances their immunosuppressive and pro-tumorigenic activities. Adoptive transfer of Gal-7-conditioned MDSCs accelerated skin carcinogenesis in *Lgals7*^*−/−*^ mice, whereas depletion of these cells using an anti-DR5 mAb delayed appearance of papillomas in *Tg46* mice, thus emphasizing the essential contribution of MDSCs to Gal-7-driven skin carcinogenesis.

Increased c-Met activation, eventually explained by the accumulation of specific mutations in its coding gene, was observed in *Tg46* papillomas. Associations between c-Met signaling and development of skin SCC [70], as well as c-Met pathway activation and Cxcl1-mediated tumor recruitment of myeloid cells have been previously described [49]. Though further mechanistic analysis should be performed, our observations in mouse papillomas together with in vitro experiments, suggest that excessive c-Met activation could be responsible for increased Cxcl1 expression and the subsequent recruitment of MDSC to TME. We might then hypothesize that overexpression of Gal-7 beyond its normal levels may favor HGF-dependent or independent c-Met signaling, which might explain the apparent increase in Cxcl1 chemokine and its contribution to recruitment of MDSCs to skin lesions. Further examination of possible intracellular mechanisms driven by Gal-7 could provide alternative explanations for this effect. In this sense, the interaction with intracellular mediators of the HGF-c-Met pathway should not be ruled out. Indeed, intracellular Gal-7 was identified as a potential mechanism by which HGF counter-represses TGF-β-stimulated profibrogenic signal transduction [71]. The fact that other cancer-driver genes in addition to c-Met showed mutations in *Tg46* papillomas, suggests that increased genomic instability might also serve as a possible mechanism implicated in this effect. In fact, a putative role of nuclear Gal-7 in DNA repair has been previously proposed [72]. In this sense, the identification of a possible interaction with PCNA, a central component of the DNA repair machinery, either directly or as a member of a molecular complex, might explain at least in part the increased mutation rate found in lesions from *Tg46* mice. Thus, if confirmed, the role of enhanced Gal-7 expression in favoring genomic instability might prevail within the intracellular compartment, complementing or synergizing with the extracellular immunoregulatory activity of this lectin, to accelerate skin carcinogenesis.

Analysis of human samples and RNAseq data support these findings, suggesting that enhanced Gal-7 expression may represent an early hallmark of human non-melanoma cutaneous malignancies. Though further studies are required, the expression levels of Gal-7 as a possible prognostic tool in NMSC management can be glimpsed. In addition, an enrichment in transcriptional programs associated with monocytic myeloid cells, higher expression of *CXCL1* -among other recruiting chemokines- as well as increased c-MET activation in lesions from AK, IEC and SCC patients compared with healthy skin, suggest that Gal-7 may trigger a regulatory circuit in human NMSC involving activation of innate immune programs in the skin TME.

In conclusion, our findings identify a Gal-7-driven pathway that impacts skin carcinogenesis by reprogramming innate immunosuppressive programs. Whereas steady-state levels of Gal-7 may control skin homeostasis [16], its heightened expression driven by sustained environmental insults may favor skin carcinogenesis. Through induction of inflammatory transcriptional programs, and eventually favoring genomic instability, Gal-7 sets up a suitable stage for tumor development, which could be further sustained by its immunosuppressive activity. Thus, targeting Gal-7 using glycan inhibitors [73], peptide antagonists [74] or specific anti-Gal-7 mAb similarly to those designed for other galectins [75] may contribute, either alone or in combination with other therapeutic strategies, to control skin carcinogenesis by reprogramming the local TME.

## Supplementary information

Supplementary Material

Dataset 1

Dataset 2

Reporting Summary

Point by point answer to reviewers

## Data Availability

All data are available within the text and Supplementary Material.
