# Supplementary material for: Galectin-7 reprograms skin carcinogenesis by fostering innate immune evasive programs
Source: Cell Death Differ. 2023 Jan 24;30(4):906–21. doi: 10.1038/s41418-022-01108-7 (PMC10070502; doi:10.1038/s41418-022-01108-7)
Supplement: Supplementary file 1 — Supplementary Material [file 41418_2022_1108_MOESM1_ESM.pdf]

Supplementary information for

Galectin-7 Reprograms Skin Carcinogenesis by Fostering Innate Immune Evasive Programs

Nicolás A. Pinto[1], Martín C. Abba[2, *], Lorena Laporte[1, *], Juan M. Pérez Sáez[1], Ada G. Blidner[1], Nicolás I. Torres[1], Rosa M. Morales[1], Sabrina G. Gatto[1], Camila A. Bach[1], Florencia Veigas[1,3], Hernán J. García Rivello[4], Peng Song[5], Jane H. Frederiksen[5], Lene Juel Rasmussen[5], Francoise Poirier[6, †], Diego O. Croci[7], Victoria Sundblad[1, *], Gabriel A. Rabinovich[1,8, *] and Juan P. Cerliani[1, *]

[1]Laboratorio de Glicomedicina, Instituto de Biología y Medicina Experimental (IBYME), Consejo Nacional de Investigaciones Científicas y Técnicas (CONICET), C1428 Buenos Aires, Argentina.
[2]Centro de Investigaciones Inmunológicas Básicas y Aplicadas (CINIBA), Facultad de Ciencias Médicas, Universidad Nacional de La Plata, C1900 La Plata, Argentina.
[3]Universidad Argentina de la Empresa (UADE). Instituto de Tecnología (INTEC). C1073 Buenos Aires, Argentina
[4]Departamento de Dermatología, Hospital Italiano, C1199 Buenos Aires, Argentina.
[5]Center for Healthy Aging, Department of Cellular and Molecular Medicine, University of Copenhagen, DK-2200, Copenhagen, Denmark.
[6]Institut Jacques Monod, UMR CNRS 7592, Paris-Diderot University, Paris, France.
[7]Instituto de Histología y Embriología de Mendoza Dr. Mario H. Burgos (IHEM-CONICET), Facultad de Ciencias Exactas y Naturales, Universidad Nacional de Cuyo, C5500 Mendoza, Argentina.
[8]Facultad de Ciencias Exactas y Naturales, Universidad de Buenos Aires, C1428 Buenos Aires, Argentina.

[*] These authors contributed equally
[†] Passed away

Running Title: Galectin-7 drives skin carcinogenesis

Keywords: Non-melanoma skin cancer; Galectin-7; Myeloid cells; Immunosuppression
Correspondence should be addressed to: G.A.R. (gabyrabi@gmail.com) or V.S. (sundbladvictoria@gmail.com).

This PDF file includes:

- **Supplementary Figures**
- **Supplementary Methods**
- **Supplementary References**

SUPPLEMENTARY FIGURES

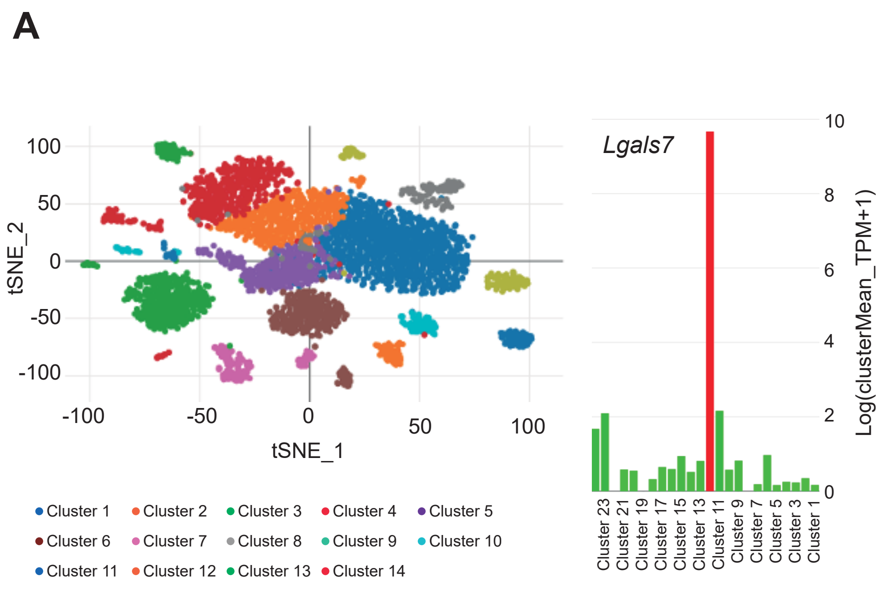

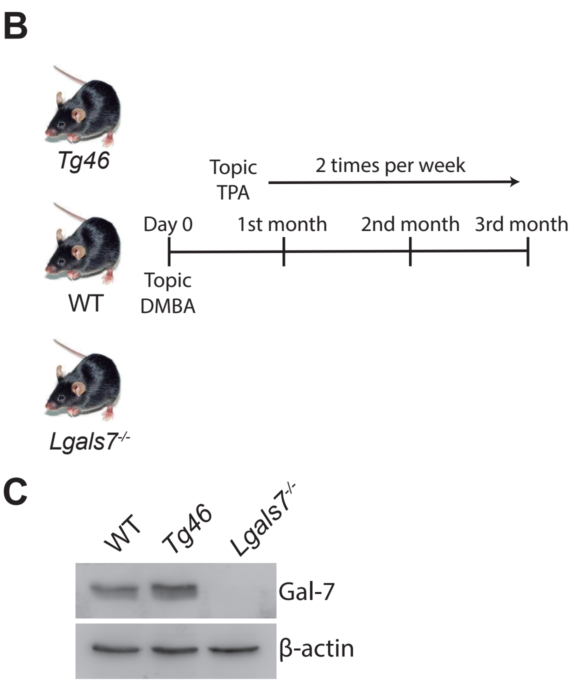

Cluster	Annotation	Gene	p_value	avg_diff	pct.1	pct.2
12-Skin	Keratinocytes	Lgals7	1.7758e-77	2.9989	0.852	0.037

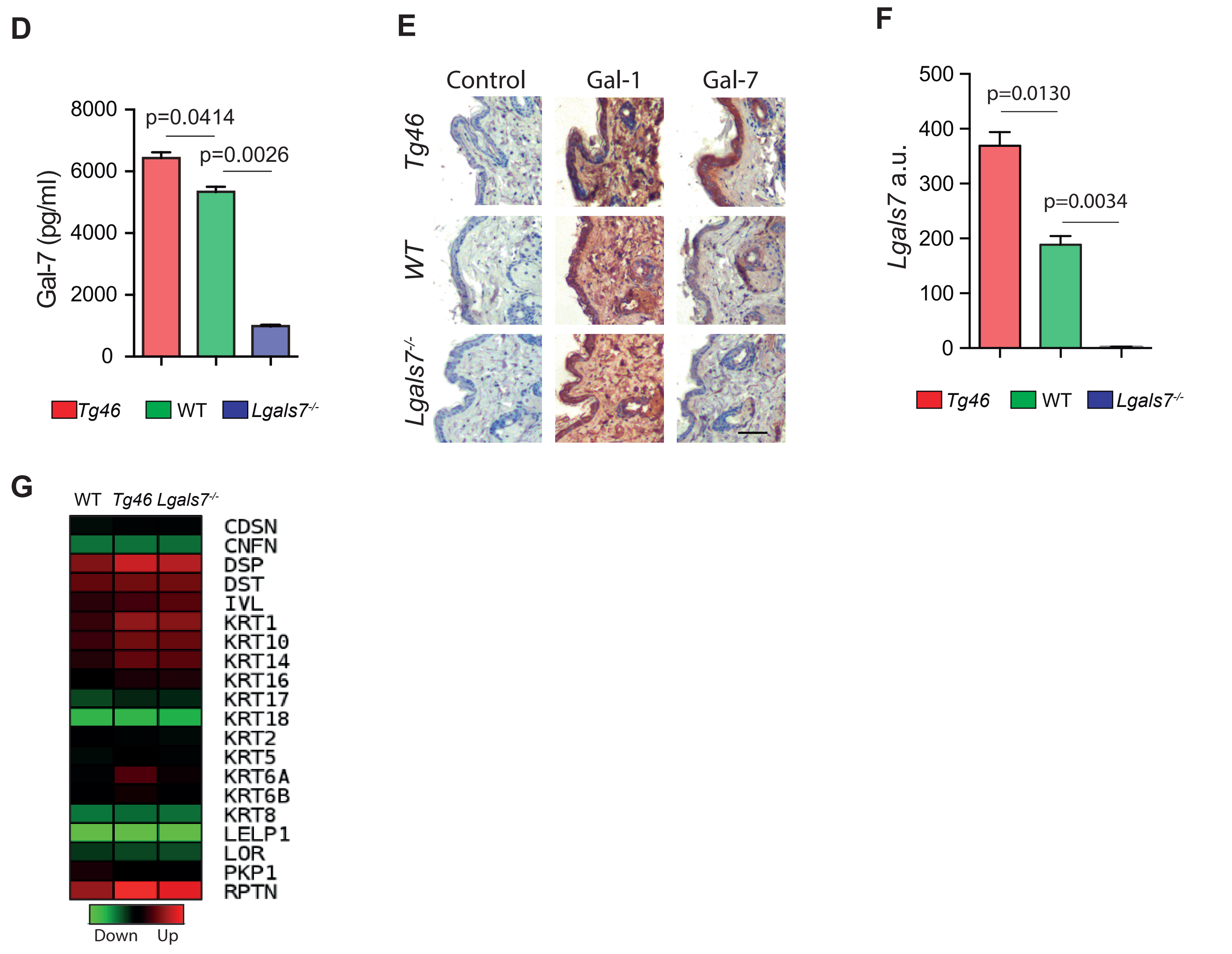

Figure S1. Gal-7 Expression in *WT*, *Lgals7*$^{-/-}$ and *Tg46* mice (Related to Figure 2)**.**

A) scRNA-seq data analysis from Mouse Cell Atlas identifies Gal-7 preferentially expressed in keratinocytes. B) *Lgals7*$^{-/-}$, *WT* and *Tg*46 mice were subjected to a two-stage carcinogenesis protocol (DMBA/TPA). C-E) Characterization of *Lgals7*$^{-/-}$, *WT* and *Tg46* mice. Western blot analysis of Gal-7 expression (C) and ELISA (D; mean ± sem; 2 independent experiments), and immunohistochemistry of Gal-7 and Gal-1 expression (E; representative images are shown, bar represents 100 µm). F) *Lgals7* expression levels in lesions from *Lgals7*$^{-/-}$, *WT* and *Tg46* mice at the end point of the carcinogenesis process.G) Heat map analysis of skin-enriched genes, obtained from RNAseq data from *Lgals7*$^{-/-}$, *Tg46* and *WT* papillomas. CDSN, Corneodesmin; CNFN, Cornifelin; DSP, Desmoplakin; DST, Dystonin; IVL, Involucrin; KRT, Keratin; LELP1, Late Cornified Envelope Like Proline Rich 1; LOR, Loricrin; PKP1, Plakophilin 1; RPTN, Repetin.

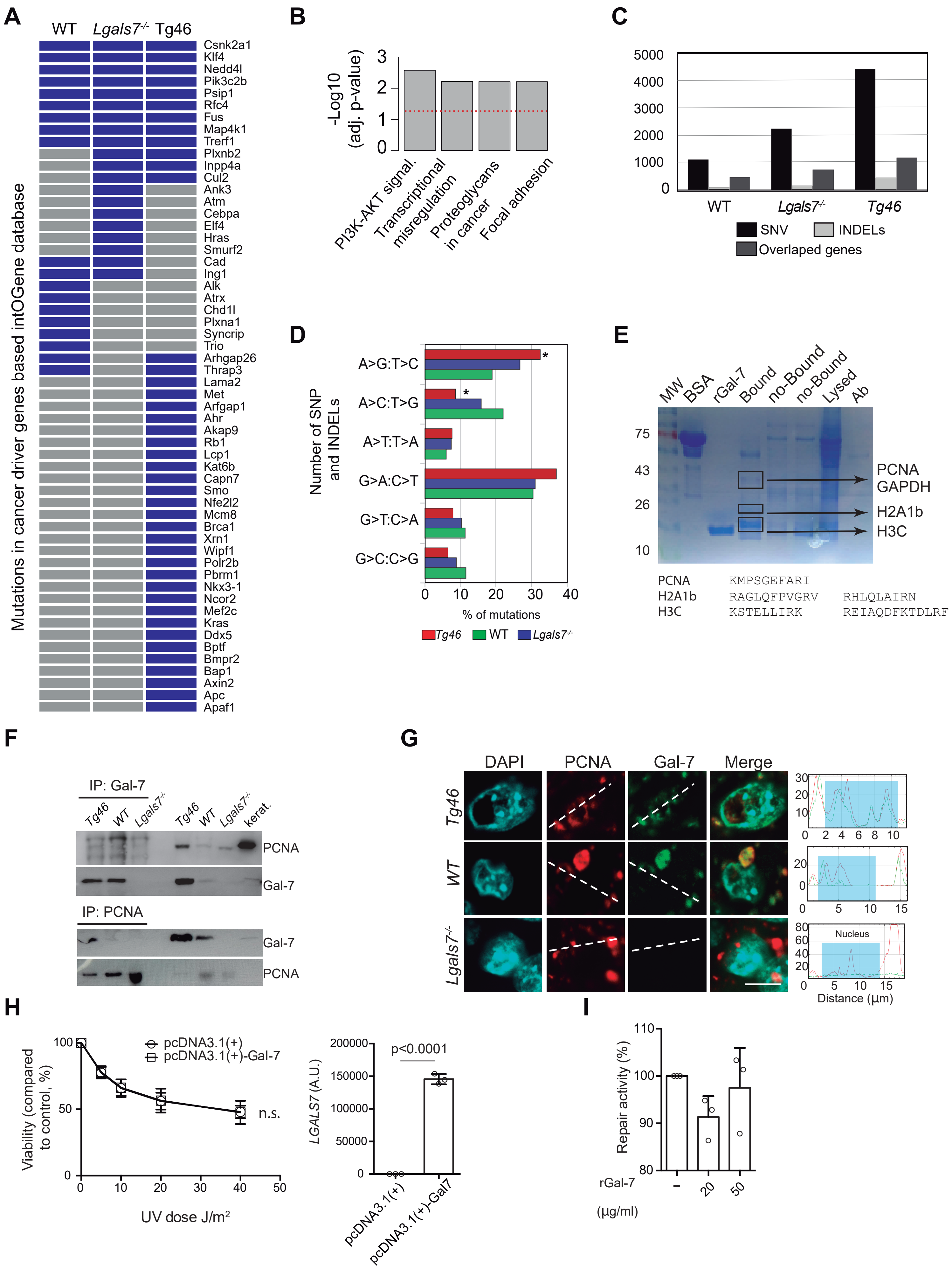

A
WT Lgals7-/- Tg46
Mutations in cancer driver genes based intOGene database
Csnk2a1
Klf4
Nedd4l
Pik3c2b
Psip1
Rfc4
Fus
Map4k1
Trerf1
Plxnb2
Inpp4a
Cul2
Ank3
Atm
Cebpa
Elf4
Hras
Smurf2
Cad
Ing1
Alk
Atrx
Chd1l
Plxna1
Syncrip
Trio
Arhgap26
Thrap3
Lama2
Met
Arfgap1
Ahr
Akap9
Rb1
Lcp1
Kat6b
Capn7
Smo
Nfe2l2
Mcm8
Brca1
Xrn1
Wipf1
Polr2b
Pbrm1
Nkx3-1
Ncor2
Mef2c
Kras
Ddx5
Bptf
Bmpr2
Bap1
Axin2
Apc
Apaf1
B
-Log10 (adj. p-value)
PI3K-AKT signal.
Transcriptional misregulation
Proteoglycans in cancer
Focal adhesion
C
WT Lgals7-/- Tg46
SNV
INDELs
Overlaped genes
D
Number of SNP and INDELs
A>G:T>C
A>C:T>G
A>T:T>A
G>A:C>T
G>T:C>A
G>C:C>G
% of mutations
Tg46 WT Lgals7-/-
E
MW BSA rGal-7 Bound no-Bound no-Bound Lysed Ab
PCNA GAPDH
H2A1b
H3C
PCNA KMPSGEFARI
H2A1b RAGLQFPVGRV RHLQLAIRN
H3C KSTELLIRK REIAQDFKTDLRF
F
IP: Gal-7
Tg46 WT Lgals7-/- Tg46 WT Lgals7-/- kerat.
PCNA
Gal-7
IP: PCNA
Gal-7
PCNA
G
DAPI PCNA Gal-7 Merge
Tg46
WT
Lgals7-/-
Nucleus
Distance (µm)
H
Viability (compared to control, %)
pcDNA3.1(+)
pcDNA3.1(+)-Gal-7
n.s.
UV dose J/m²
LGALS7 (A.U.)
p<0.0001
pcDNA3.1(+)
pcDNA3.1(+)-Gal7
I
Repair activity (%)
rGal-7
(µg/ml)

Figure S2. A-D) Exome-seq analysis and mutational profiles of papillomas (n=3 pooled tumors per group) from *Lgals7*$^{-/-}$, *WT* and *Tg46* mice subjected to carcinogenesis protocol. A) Missense mutations in cancer driver genes identified in each tumor type (Blue squares) B) Barchart of bioprocesses found to be enriched according to the mutated cancer driver genes that were identified. C) Incidence of different type of DNA mutations. D) Frequency of different single-base substitutions in each tumor type (*$p<0.05$) compared using the chi-square test. E-I) Association of intracellular Gal-7 with PCNA. E) Identification of PCNA as a Gal-7 interaction partner. Immunoprecipitated complexes obtained from pulldown experiments were separated by polyacrylamide gel electrophoresis. Bands (upper panel) corresponding to proteins further identified through mass spectrometry analysis (lower panel) are shown. F-G) Evaluation of Gal-7-PCNA interaction in papillomas from *Tg46*, *WT* and *Lgals7*$^{-/-}$ mice. F) Co-immunoprecipitation of total protein extracts. G) Representative images of immunofluorescence staining (left panel; Gal-7, green and PCNA, red; bar represents 10 μm) and representative plots of intracellular localization of Gal-7 and PCNA (right panel) are shown. H-I) Evaluation of PCNA activity in the presence of increased Gal-7 expression. H) Quantification of NER activity, as evaluated by sensibility to UV radiation. Viability of Hela cells transfected with pcDNA3.1(+)-Gal7 or with empty vector (pcDNA3.1(+)) and irradiated with UV light (left panel; mean ± sem; 3 independent experiments) and *LGALS7* expression levels in Hela cells transfected with each plasmid, evaluated by qRT-PCR (right panel; mean ± sem; 3 independent experiments) are shown. I) Evaluation of *in vitro* MMR activity, in the presence or absence of rGal7 (mean ± sem; 3 independent experiments).

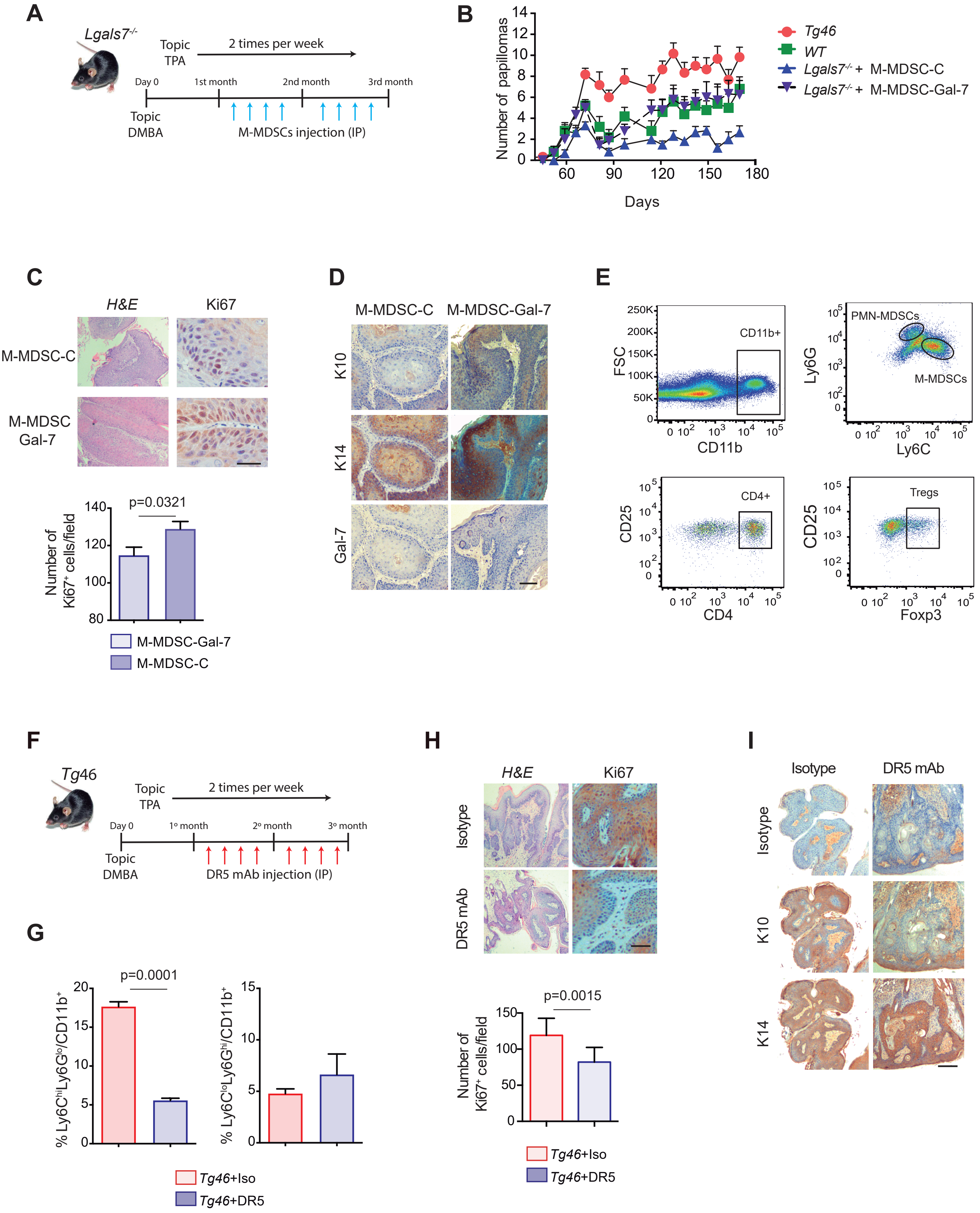
A
Lgals7-/-
Topic TPA
2 times per week
Day 0
1st month
2nd month
3rd month
Topic DMBA
M-MDSCs injection (IP)
B
Number of papillomas
Days
Tg46
WT
Lgals7-/- + M-MDSC-C
Lgals7-/- + M-MDSC-Gal-7
C
H&E
Ki67
M-MDSC-C
M-MDSC Gal-7
p=0.0321
Number of Ki67+ cells/field
M-MDSC-Gal-7
M-MDSC-C
D
M-MDSC-C
M-MDSC-Gal-7
K10
K14
Gal-7
E
FSC
CD11b+
CD11b
Ly6G
PMN-MDSCs
M-MDSCs
Ly6C
CD25
CD4+
CD4
Tregs
Foxp3
F
Tg46
Topic TPA
2 times per week
Day 0
1º month
2º month
3º month
Topic DMBA
DR5 mAb injection (IP)
G
p=0.0001
% Ly6ChiLy6Glo/CD11b+
% Ly6CloLy6Ghi/CD11b+
Tg46+Iso
Tg46+DR5
H
H&E
Ki67
Isotype
DR5 mAb
p=0.0015
Number of Ki67+ cells/field
Tg46+Iso
Tg46+DR5
I
Isotype
DR5 mAb
Isotype
K10
K14

Figure S3. MDSCs Mediate Gal-7-Driven Skin Carcinogenesis (Related to Figure 7). A-E) M-MDSCs differentated from *WT* BM-derived cells and activated in the presence (M-MDSC-Gal-7) or absence (M-MDSC-C) of rGal-7 were injected weekly in *Lgals7*$^{-/-}$ mice, during the two-stage carcinogenesis protocol. A) Experimental design. B) Tumor growth weekly monitored and scored, in *Lgals7*$^{-/-}$ mice treated with M-MDSC-Gal-7 or M-MDSC-C, and in *WT* and *Tg46 animals*. At each time point, data are expressed as average of tumor number for each experimental group (mean ± sem; 2 independent experiments). C) Analysis of Ki67^{+} cells in papillomas from *Lgals7*$^{-/-}$ mice treated with M-MDSC-Gal-7 or with M-MDSC-C, evaluated by immunohistochemistry. Representative images (upper panel; bar represents 100 µm) and quantification of Ki67^{+} cells (lower panel; mean ± sem) are shown. D) K10, K14 and Gal-7 expression in papillomas from *Lgals7*$^{-/-}$ mice injected with M-MDSC-C or with M-MDSC-Gal-7, evaluated by immunohistochemistry. Representative images are shown (bar represents 100 µm). E) Characterization of tumor-associated infiltrate. Representative dot plots of PMN-MDSCs, M-MDSCs, and CD4^{+} CD25^{+} Foxp3^{+} cell frequencies in papillomas from *Lgals7*$^{-/-}$ animals. F-I) *Tg46* mice were weekly treated with anti-DR5 mAb or with isotype control mAb beginning from the first month of the carcinogenesis protocol (n=3, 5, 6 animals per group; 3 independent experiments). F) Experimental design. G) Frequency of M-MDSCs and PMN-MDSCs in the spleen of *Tg46* mice after treatment with anti-DR5 or isotype control mAb (mean ± sem of 3 independent experiments). H) Analysis of Ki67^{+} cells in papillomas from both experimental groups, evaluated by immunohistochemistry. Representative images (upper panel; bar represents 100 µm) and quantification of Ki67^{+} cells (lower panel; mean ± sem) are shown. I) K10 and K14 expression in papillomas from *Tg46* mice injected with anti-DR5 or with isotype control mAb, evaluated by immunohistochemistry. Representative images are shown (bar represents 100 µm).

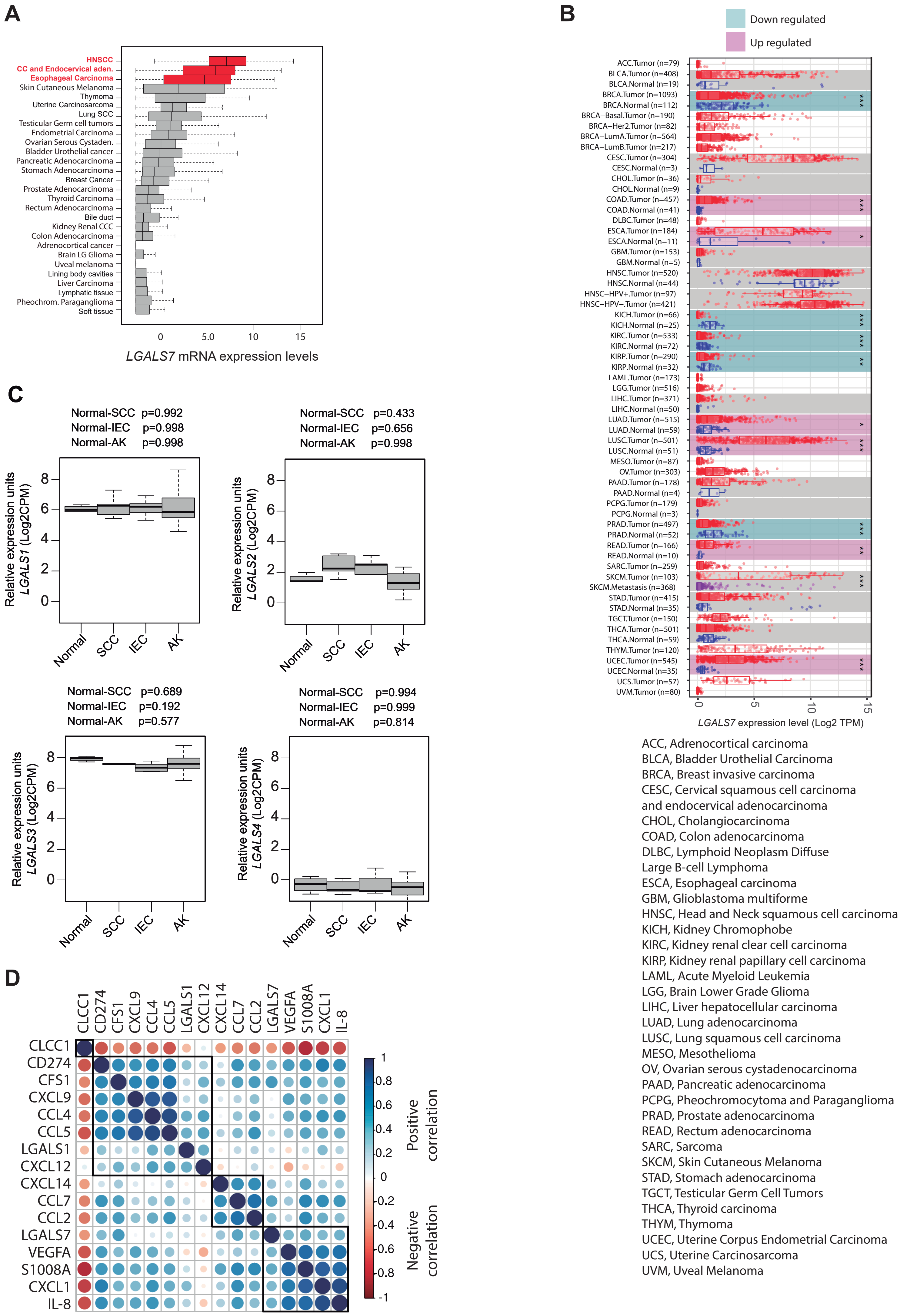

ACC, Adrenocortical carcinoma
BLCA, Bladder Urothelial Carcinoma
BRCA, Breast invasive carcinoma
CESC, Cervical squamous cell carcinoma and endocervical adenocarcinoma
CHOL, Cholangiocarcinoma
COAD, Colon adenocarcinoma
DLBC, Lymphoid Neoplasm Diffuse Large B-cell Lymphoma
ESCA, Esophageal carcinoma
GBM, Glioblastoma multiforme
HNSC, Head and Neck squamous cell carcinoma
KICH, Kidney Chromophobe
KIRC, Kidney renal clear cell carcinoma
KIRP, Kidney renal papillary cell carcinoma
LAML, Acute Myeloid Leukemia
LGG, Brain Lower Grade Glioma
LIHC, Liver hepatocellular carcinoma
LUAD, Lung adenocarcinoma
LUSC, Lung squamous cell carcinoma
MESO, Mesothelioma
OV, Ovarian serous cystadenocarcinoma
PAAD, Pancreatic adenocarcinoma
PCPG, Pheochromocytoma and Paraganglioma
PRAD, Prostate adenocarcinoma
READ, Rectum adenocarcinoma
SARC, Sarcoma
SKCM, Skin Cutaneous Melanoma
STAD, Stomach adenocarcinoma
TGCT, Testicular Germ Cell Tumors
THCA, Thyroid carcinoma
THYM, Thymoma
UCEC, Uterine Corpus Endometrial Carcinoma
UCS, Uterine Carcinosarcoma
UVM, Uveal Melanoma

Figure S4. Gal-7 Expression in Human Tumors (Related to Figure 8). A, B) *LGALS7* gene expression levels in different human tumors (9,186 primary tumors, classified according to tumor type) from the TCGA-PANCAN dataset (data obtained from cBioPortal). *LGALS7* mRNA expression in different human cancer types (A), and in primary tumors relative to its expression in normal adjacent samples (B) are shown. *p<0.05; **p<0.01; ***p<0.001. C) Assessment of *LGALS1* (upper left panel), *LGALS2* (upper right panel), *LGALS3* (lower left panel) and *LGALS4* (lower right panel) gene expression in normal and NMSC human samples from E-MTAB-5678 dataset. D) Expression levels correlation map of chemokines in human NMSC analyzed from RNAseq data (E-MTAB-5678).

SUPPLEMENTARY METHODS

Cell Lines and Culture

Human KCs (HaCaT; cell lines service #300493) and mouse KCs (Kera-308; cell lines service #400429) were grown in DMEM (Gibco) containing 10% fetal bovine serum (FBS; Gibco) and 1% antibiotic-antimycotic cocktail (Gibco). Cells were grown on plastic culture dishes at 37°C in a humidified incubator with 5% CO_2. When 80% of confluence was reached, cells were exposed to different treatments including DMBA, TPA, UVB light, HGF (10 ng/ml; Prepotech) or control medium for 16 h.

Conditioned media from murine KC for migration assays were produced as follows. Kera-308 were plated in p60 dishes until 80% confluence. Cells were treated with HGF (10 ng/ml) at 37°C for 30 minutes, or with anti-CXCL1 blocking antibody (0.25 µg/mL, R&D MAB453) for 30 minutes prior to the addition of 10 ng/ml rHGF, or remained untreated (control). Cells were washed with PBS and fresh media was added. After 16 hs, the media was collected.

Preparation of Epidermal Cell Suspensions

Epidermal cell suspensions were prepared from shaved back skin or from ear skin. Briefly, mice ear was separated in two parts so as to place both inner and outer sides of the ear, placing the skin on its dermal side-down. Then, ears fragments were incubated with 12,5 mg/ml dispase II (Roche) for 2 h at 37°C. Dorsal skin samples (dermal side-down) were also incubated with 4.8 mg/ml dispase II (Roche) for 2 h at 37°C. In both cases, the epidermal layer was then carefully separated from the dermis and further digested with TrypLE Express solution (Gibco) for 15 min at 37°C. Single cell suspensions were filtered with a 70 µm mesh, washed with PBS and resuspended in corresponding buffers or medium for subsequent analysis.

Primary Cultures of Mouse Keratinocytes

Epidermal cell suspensions were prepared as described above. Then, KCs were seeded in p60 plates and cultured in KC medium (Gibco) supplemented with mouse KC growth

supplement (Gibco) and antibiotics/antimycotics. At confluence, KC primary cultures were starved for 12 h and incubated with TPA (16 nM) or DMBA (64 µM) for 18 h, exposed to UVB irradiation (average dose of 10 J/m^2) for 2 min or incubated with HGF (20 ng/ml; Prepotech) for 12 h. Culture medium was collected, and KCs were processed with lysis buffer or Trizol (Invitrogen).

Inmunohistochemistry

Skin samples from mice ears or from back skin were fixed in 4% formalin and embedded in paraffin. Slides (5 µm) were dewaxed and rehydrated in serial ethanol dilutions. After antigen retrieval with sodium citrate solution (pH 6.0, 100°C for 15 min), endogenous peroxidase activity was blocked with 3% H_2O_2 treatment and non-specific binding was blocked with 10% FBS in PBS. Samples were incubated for 16 h at 4°C with primary antibodies: anti-Gal7 (ab10482, Abcam), anti-Gal7 (ab138513, Abcam), anti-Ki67 (14-5698-82, eBioscience), anti-CKT10 (904301, Biolegend),CKT14 (905304, Biolegend), anti-CD11b (PA5-79532, ThermoFisher), anti-PCNA (sc-25280, Santa Cruz), anti-DR5 (BE0161, BioXcell), anti-CXCL1 (PA5-86508, Invitrogen), anti-cMet (PA5-85951, Invitrogen), and anti- phospho-c-Met (44-882G, Invitrogen). Biotinylated secondary antibodies and streptavidin/HRP complexes were used following the manufacturer's instructions (Vectastain kit, Vector Labs). Sections were treated with diaminobenzidine (DAB, Dako) or 3-amino-9-ethylcarbazole (AEC, Vector laboratories) chromogenic substrate, counterstained with haematoxilin, dehydrated and mounted in xylene-based mounting medium (Life Sciences). Photographs were taken at 40x, 100x and 400x magnifications with an Olympus CX31 microscope. At least 7 fields were analyzed for cell number and staining intensity. Intensity was automatically quantified using Photoshop software.

Real-time Quantitative RT-PCR

SYBR Green PCR Master Mix (Applied Biosystem) was used with an ABI PRISM 7500 Sequence Detection Software (Applied Biosystem). Primers used were:

Mouse *Cxcl1* Fw: 5' –ACCGAAGTCATAGCCACACTC- 3', Rv: 5' –CTCCGTTA CTTGGGGACACC- 3'; mouse *Ccl7* Fw: 5'- CCCTGGGAAGCTGTTATCTTCAA -3', Rv: 5'-CTCGACCCACTTCTGATGGG -3'; mouse *VEGFa* Fw: 5′-CGGGCCT

CGGTTCCAG– 3′, Rv: 5′ -CTGGGACCACTTGGCATGG– 3′; mouse *S1008A* Fw: 5′-TTCAAGACATCGTTTGAAAGGAAA– 3′, Rv: 5′ -AGGTTGCT CAAGGCCTTCTC – 3′; mouse c-*Met* Fw: 5′- TGGCTACTGCTCTGGAGGAC- 3′, Rv: 5′-AGGAGTT GATCACATGCCAAGC-3′; mouse *Lgals7* Fw: 5′- TTCCACGTGAACCTGCTGT -3′, Rv: 5′-GAAAGTGGTGGTACTGTGCG -3′.

Western Blot Analysis

Total protein extracts from ear skin or back skin samples from *in vivo* experiments or from KC primary cultures were prepared by cell lysis in ice-cold lysis buffer (50 mM Tris-HCl pH 7.5, 150 mMNaCl, 20 mM EDTA, and 1% Nonidet P-40) supplemented with a mixture of protease inhibitors (Sigma-Aldrich). Equal amounts of protein extracts (30 µg) were denatured, separated by SDS-PAGE and transferred onto a nitrocellulose membrane (GE Healthcare Life Sciences). After saturation of nonspecific binding sites by incubating for 1 h in TBS containing 20 mM Tris-HCl, 0.1% Tween 20 and 5% nonfat dry milk, membranes were incubated with primary antibodies raised against Gal-7 (ab138513, Abcam), GAPDH (sc-47724, Santa Cruz), c-Met (PA5-85951, Invitrogen), phospho-c-Met (44-882G, Invitrogen), followed by incubation with the appropriate HRP-conjugated secondary antibodies (Vector Labs) and processed by chemoluminiscence (Millipore).

Exome-Seq Analysis

DNA from mouse skin papillomas (3 pooled tumors for each genotype; *WT*, *Lgals7*$^{-/-}$ and *Tg46*) were purified using the DNeasy Blood and Tissue Kit (Qiagen). Only DNA samples with 260/280 absorbance ratios greater than 2.0 were processed for library construction using the SureSelect Mouse All Exon Kit (Agilent Tech) as enrichment system, covering ~ 49.6 Mbp of target sequence. Seventy-five nt paired-end sequencing was performed using an Illumina HiSeq-2500 platform at MedGenome Core Facility. Image analysis, base-calling and error calibration were performed using Illumina's Genome analysis pipeline. Sequencing was performed reaching an average depth of 200X per sample. The paired-end reads were aligned to the reference mouse genome primary assembly GRCm38/mm10 release downloaded from Ensembl database using BWA (v0.7.15) and marked for duplicates using Picard tool (v1.115) Sort Sam command. Duplicate reads were removed using Picard MarkDuplicates command. Subsequently,

SNVs and short INDELs were identified using GenomeAnalysisTKLite-2.3-9 toolkit. For realignment and base-recalibration the dbsnp138 variants were used. The identified variants were further filtered and 'only passed' and 'on-target' variants were considered for further analysis. The 'on-target' were calculated based on the coordinate of the target regions provided. The identified variants were annotated using a MedGenome in-house program (VariMAT) based on the Ensembl gene model 78 release. The variant class prediction was done using VeP Release 78 (http://www.ensembl.org/Tools/VEP). Detected mutations were further filtered by focusing only on those genes considered cancer driver genes, as defined by the IntOGene mutational cancer drivers database (https://www.intogen.org/search).

Transfection and Cell Viability Assays

HeLa cells were maintained in DMEM (10% FBS and 1% penicillin-streptomycin) at 37°C and 5% CO_2. Cells were trypsinized and seeded in 6-well plates (300,000 cells/well). After 24 h, cells were transfected with 1.2 µg hGal-7_pcDNA3.1(+) or pcDNA3.1(+) (GenScript) using PolyJet transfection reagent (SL100688, SignaGen Laboratories) with duplicates. Twenty-four h after transfection, total RNA was extracted following Direct-zol™ RNA MiniPrep Plus protocol (BioSite-R2070, Nordic Biosite), and cDNA was obtained using High-Capacity cDNA Reverse Transcription Kit (10400745, Fisher Scientific) following manufacturer's instructions. Expression of Gal-7 was confirmed by real time qPCR. Cells were trypsinized and seeded in 96-well plates (10,000 cells/well). Forty-eight h after transfection, cells were exposed to different doses of UVB irradiation. After an additional 24 h-incubation period, cell viability was determined by MTS assay (G3580, Promega).

In Vitro DNA Mismatch Repair Assay

The *in vitro* MMR assay was performed as described previously (Drost et al., 2013). HeLa cell nuclear extract was prepared using CelLytic™ NuCLEAR™ Extraction Kit (NXTRACT-1KT, Sigma-Aldrich), following manufacturer's instructions. Extracts (75 µg) were incubated with 100 ng of G/T substrate at 37°C for 30 min, in 25 µl reactions containing 20 mM Tris-HCl (pH 7.6), 1.5 mM ATP, 0.1 mM of all four dNTPs and 5 mM $MgCl_2$. Controls included HeLa cell nuclear extract alone, while test reactions contained 20 or 50 µg/ml rGal-1 or rGal-7. Reactions were terminated by addition of 50 µl stop

solution (50 mM EDTA, 2% SDS, and 2 mg/ml proteinase K). Mixtures were further incubated at 37°C for 45 min. The following steps were performed as described (Drost et al., 2010). In brief, DNA was purified from reaction mixture by MinEluteReaction Cleanup Kit (28206, Qiagen). Restriction digestions were carried out in 12 µl reactions containing NlaIII endonucleases (R0125S, NEB), for 60 min at 37°C. Two µl of digested substrate was mixed with 8 µl Hi-Di formamide containing 0.25 µl GeneScan™ 500 ROX™ dye size standard (401734, Thermo Fisher Scientific-Life Tech) and fragment analysis was performed on a 3130 DNA analyzer (Applied Biosystems). Data was analyzed using GeneMarker V1.5 software. Repair levels were calculated by dividing the height of the MMR-specific peak by the total fluorescent signal.

Pulldown of Gal-7 Complexes and Immunoprecipitation

Intracellular protein extracts (500 µg) were incubated with rGal-7 (50 µg/ml) for 30 min and immunoprecipitated as described below. Gal-7 complexes were separated in 12/15% SDS PAGE and bands were analyzed by mass spectrometry (LANAIS PROEM, University of Buenos Aires, Argentina). Intracellular protein extracts (500 µg) from *WT*, *Lgals7*$^{-/-}$ and *Tg46* tumors were incubated with 2 µg of anti-Gal-7 polyclonal antibody (ab10482; Abcam). Immunocomplexes were captured with protein G PLUS-Agarose (Santa Cruz). The immunoprecipitated complexes were separated by a 12/15% SDS-PAGE, transferred onto nitrocellulose membranes and probed with anti-PCNA and anti-Gal-7 antibodies.

SUPPLEMENTARY REFERENCES

- Drost M, Zonneveld J, van Dijk L, Morreau H, Tops CM, Vasen HF, et al. A cell-free assay for the functional analysis of variants of the mismatch repair protein MLH1. Hum. Mutat. 2010; 31,247-253.
- Drost M, Lützen A, van Hees S, Ferreira D, Calléja F, Zonneveld JB, et al. Genetic screens toidentify pathogenic gene variants in the common cancer predisposition Lynch syndrome. Proc.Natl. Acad. Sci. U S A. 2013;110, 9403-9408.
