# Supplementary material for: Galectin-7 reprograms skin carcinogenesis by fostering innate immune evasive programs
Source: Cell Death Differ. 2023 Jan 24;30(4):906–21. doi: 10.1038/s41418-022-01108-7 (PMC10070502; doi:10.1038/s41418-022-01108-7)
Supplement: Supplementary file 5 — Point by point answer to reviewers [file 41418_2022_1108_MOESM5_ESM.docx]

**POINT-BY-POINT RESPONSE TO REVIEWERS' COMMENTS
Answers to Reviewer #1**

We thank to the reviewer for his/her detailed analysis of our manuscript. Suggestions were of great help and were taken into consideration. New experiments were performed to address his/her concerns, which overall helped to strengthen our study.

Please find below a point-by-point response to each individual comment:

*1. In the abstract section, the authors should provide define DMBA/ TPA (carcinogens DMBA/ TPA)*

Following the reviewer´s suggestion, we have defined DMBA/TPA in the abstract section (lines 44 to 46 of the revised version).

2. *For the western blot in figure 1C is of poor quality. From the present one it is difficult to appreciate significative increase of GAL7 signal in the 4th lane respect to the control.*

Preparation of epidermal cell suspensions is a laborious issue and protein extracts are likely to contain extracellular matrix components that hinder electrophoresis. Despite several attempts to improve the quality, western blots quality was not as good enough as desired. Regardless of these technical details, though an increase in Gal7 signal is hard to appreciate with a naked eye, when we relativized to GAPDH signal, a significant increase is observed in each condition, including UVB treatment. Additionally, the assessment of Gal7 expression in response to genotoxic and inflammatory insults is complemented with immunofluorescence staining of mouse skin and is also evaluated in the HaCaT cell line by immunofluorescence staining and Western blot analysis (revised Figure 1).

*3. For figure 1E, provide quantification of GAL 7 signal and provide a quantitative western blot in addition to the immunofluorescence.*

Following the reviewer suggestion, we have included quantification of Gal7 signal by immunofluorescence (Revised Figure 1 E). In addition, Gal7 expression in HaCaT upon exposure to insults was also evaluated by Western blot (Revised figure 1 F)

*4. In figure 2E, provide quantification of K10, K14 and GAL7 signals. As the authors do for KI67 signal.*
Following the reviewer suggestion, quantification of K10, K14 and Gal7 signals was included in the revised version (Revised Figure 2). In order to increase the number of images per animal included in the quantification, additional IHC were performed. Notably, K10 expression was found to be reduced in *Tg46* mice, compared to WT and *Lgals7^-/-^*animals. This new observation has been incorporated in the revised version (lines 310 to 311).

*5. For figure2, the authors should provide the list of differentially expressed genes in TG46, WT and GAL7-/- mice skin papillomas.*

Following the reviewer suggestion, the list of differentially expressed genes (DEG) in skin papillomas from *Tg46* vs *WT* mice, and in lesions from *Lgals7^-/-^*mice vs those from *WT* animals were included as Supplementary File RNAseq (xlsx.file), and mentioned in line 320.

*6. Page 14, line 289, what does it mean “cell populations in dLN”? Maybe draining Lymph nodes? The authors should provide extensions for the acronymous that were mentioned for the first time (e.g. TME at line 297).*

For clarity, we have now defined dLN when first used in Results section (line 340), and in Materials and Methods (Flow Cytometry section). In addition, we have included the acronymous extension of tumor microenvironment (TME) in line 349 of the revised version. We apologize for the oversight.

*7. Moreover, in the same chapter, the authors should explain what the immunophenotypic profile CD11b+Gr1+ corresponds to, for readers who don’t work in the field of immunology.*

Following the reviewer´s suggestion we have included a short explanation on the immunophenotype of these cells in the revised version (lines 338-339).

*8. Same comment for “CD4+CD25+Foxp3+ cells or in the CD8+/CD4+CD25+Foxp3+ 293 ratio”. What is this ratio indicative of?*

We have also included a brief explanation on these markers and the significance of this ratio in the revised version (lines 344 to 346)

*9. For figure 3G the authors should provide statistical analysis.*

We apologize for the oversight. We have now provided statistical analysis of the results shown in Figure 3G.

*10. In figure 3 the authors should use the same font size and align the charts. Same comment for charts in figure 2c and in general for all the figures.*

We have reviewed the typography, font size and design details of all the figures trying to give uniformity to all the figures.

*11. In the fourth panel of figure 3H, y axis is missing.*

Y axis label was included in the revised version. We apologize for the oversight.

*12. From figure 4B it seems that WT and not Tg46 papilloma have more Cxc1 cells. This needs to be carefully evaluated….*

The number of Cxcl1^+^ cells in papillomas from different groups of mice was carefully evaluated and quantified, analyzing 10 imagines per group, for 3 independent experiments with N=5 mice per group in each experiment. For clarity and better representation of the results, a new image representative of the WT group was selected and replaced in the revised figure.

13*. Results described in figure 4C and 4D are very interesting. From them, it seems that either GAL7 knock down or overexpression in particular, drive the accumulation of mutations in genes encoding for oncogenic drivers. The authors should better discuss this set of results. A higher number of PCNA-positive cells in TG46 mice compared to WT mice, can be explained by increased proliferation rate, parallel to increased KI67 and does not explain genomic instability. In fact, in vitro assays suggest that neither NER, nor mismatch repair are affected by increased Gal7 levels. May the authors search for the differential expression of genes related to genomic instability and DNA repair pathways in RNA-seq data from WT, GAL7-/- and TG46? It is not clear why in figure 4C the* *authors list only mutations of the BRCA and c-met loci. Would it be possible to have a more inclusive table with mutations also at other genes listed in figure 4D?*

We agree with the reviewer that results on the accumulation of mutations are very interesting, particularly in papillomas overexpressing Gal7. Indeed, we have included an extended list of cancer-driver gene mutations in the revised Supplementary Figure 2 (panel A). Among them, we decided to focus particularly in *BRCA1* and *c-Met* genes, due to the relevance of their biological function in the context of our study, and to the variety of mutations found in each locus.

Association of aberrant c-Met signaling and cancer progression has been extensively reported [1, 2]. In addition, previous evidence show association between c-Met pathway activation and Cxcl1-mediated recruitment of myeloid cells to the TME [3]. Given that tumors overexpressing Gal-7 (*Tg46*) showed significantly higher expression of Cxcl1 and increased infiltration of myeloid cells, we decided to focus our attention particularly on the c-Met pathway. We speculated that abnormal c-Met expression or activation could be responsible for increased Cxcl1 expression and the consequent recruitment of MDSC to the TME. In this context, the high variability of genomic missense mutations identified in c-Met loci added interest to our approach.

Regarding the Brca1 gene, based on the role of its encoded protein in maintaining genomic stability, the association of mutations with a higher risk of cancer (specially breast and ovarian, but also colorectal and others), and our findings demonstrating mutations in Brca1 in Gal-7-overexpressing *Tg46* papillomas, study of this gene becomes particularly interesting. Nevertheless, for clarity, mutations in BCRA1 were only mentioned in the text but not described in detail in Figure 4 in order to focus in the c-Met gene, in this particular study.

Though only mutations in c-Met genes are detailed in Figure 4, the complete mutational profile of each type of tumor was included as a supplementary file in the revised version (Supplementary Data Mutational Profiles)

Regarding PCNA, in view of the important accumulation of mutations in papillomas overexpressing Gal-7, we aimed at seeking for possible mechanisms that could eventually explain genomic instability. In this context, we developed pull-down experiments followed by mass spectrometry analysis, to evaluate possible intracellular molecular partners that might be interacting with Gal-7. In this particular case, proliferating cell nuclear antigen (PCNA), an essential protein that participates in a variety of processes of DNA metabolism, including DNA replication and repair, chromatin organization and transcription and sister chromatid cohesion, has been identified as a possible Gal-7-binding partner.

Thus, we decided to evaluate if any of the DNA repair mechanisms in which PCNA is known to be involved could be altered by its possible interaction with Gal-7. In our setting, neither nucleotide excision repair (NER), the main pathway used by mammals to remove bulky DNA lesions such as those formed by UV light and environmental mutagens, nor mismatch repair mechanism were affected by the increased levels of Gal-7. Nevertheless, alteration in other DNA repair mechanisms, such as translesion DNA synthesis (TLS), cannot be ruled out. Indeed, post-translational modifications of PCNA play important roles in controlling DNA damage tolerance pathways, which include TLS and template switching. For example, TLS is mediated by mono-ubiquitination of PCNA while the template switching pathway is promoted by poly-ubiquitination of PCNA. Simultaneous mono-ubiquitinations on multiple units of the PCNA homotrimeric ring promote an unidentified damage tolerance mechanism that remains to be fully characterized. Furthermore, SUMOylation of PCNA in mammalian cells can negatively regulate recombination [4]. Alternatively, other PCNA functions, not related to DNA repair, might be affected by Gal-7 overexpression. Moreover, the possibility that PCNA-independent mechanisms could be affected by Gal-7 overexpression, in which case the interaction between Gal-7 and PCNA would not be related to increased mutations, cannot be ruled out either.

In any case, given the relevant role of PCNA not only in several DNA repair mechanisms, but also as essential factor in DNA replication, its expression could be considered a standard indicator of proliferation, used to assess the growth fraction of a given cell population. Thus, we agree we the reviewer that a higher number of PCNA-positive cells in Tg46 mice compared to WT mice, could be explained by an increased proliferation rate. Consequently, for clarity, quantification of PCNA-positive cells was not included in the revised Supplementary Figure 2.

We recognize that the biological relevance of PCNA-Gal-7 interaction should be further analyzed in future studies, as well as the possible implications of this molecular interaction with either DNA replication functions or with DNA repair mechanisms.

Regarding a possible pattern of differentially expressed genes, associated with genomic instability and DNA repair pathways in RNA-seq, expression of Primpol (which encoded protein is responsible of tolerance to DNA damage by mediating uninterrupted fork progression after UV irradiation and reinitiating DNA synthesis) was significantly increased (p=0.0027), while expression of RAD50 (encoding a double strand break repair protein) was significantly decreased (p=0.0352) in papillomas from Tg46 mice, compared to those from WT animals. The complete lists of DEG in papillomas from Tg46 mice versus WT, and from KO animals versus WT mice was included as a Supplementary file in the revised version (Supplementary File RNAsq).

14. *In page 16, the authors should introduce better the c-Met-Cxcl1 axis. On what basis the authors link c-met with Cxcl1 cells?*

Following the reviewer´s suggestion, in the revised version we have made our best to clarify the link between c-Met activation and CXCL-1 expression. Previous evidence documenting the link between c-Met activation and CXCL-1 expression and its impact in cancer survival and metastasis is mentioned in lines 357-361. Additionally, following results related to exome seq analysis and seeking for possible causes of c-Met aberrant signaling, from line 381 to 396, we discuss again this topic highlighting previous studies, and showing new results demonstrating: a) increased phosphorylation of c-Met in tumors, but not in normal skin of *Tg46* compared with WT mice, and in WT compared to *Lgals7^-/-^*mice, b) a considerable increase in *Cxcl1* expression in KCs upon HGF (c-Met ligand) stimulation, showing that c-Met activation may indeed control *Cxcl1* expression in KCs; c) a trend toward an increased migration of myeloid cells when cultured with supernatant from HGF-treated KT, compared to that from untreated KTs, and reduction of this effect in the presence of anti-Cxcl1 blocking antibody. Altogether, these results suggest a link between c-Met activation and Cxcl1 expression, which might contribute, at least in part, to recruitment of myeloid cells to TME.

The present study highlights, for the first time the pivotal role of Gal-7 in skin carcinogenesis in a broadly fashion, unfolding several observations that will be dissected in more detail in future studies. Among them, the impact of c-Met-dependent CXCL-1 expression on MDSC recruitment is a very interesting topic which deserves a more in-depth study. However, given the number of immune and non-immune observations arising during this study, we decided to focus on the role of Gal-7 on MDSC biology and the impact of this interaction on tumor development. We plan to continue exploring on other emerging topics from this work, and to deep insight the mechanisms underlying MDSC recruitment to the TME.

*15. In the same page, line 334, the authors found increased phosphorylation of c-Met in tumors (Figure 4H), but not in normal skin (data not shown), of Tg46 mice. In my opinion, if they state that the increased phosphorylation of c-Met does not occur in normal skin, they should show the result, otherwise they can remove the sentence “but not in normal skin” from the text.*

We agree with the reviewer´s comment. Consequently, we have included an additional panel in Revised Fig 4 (panel F) showing c-Met phosphorylation levels in normal skin and mentioned it in the revised manuscript (lines 386-387).

16.*From line 333 to line 342 the authors only perform correlative analyses e.g tumors with increased c-met phosphorylation show increased cxcl1+ cells and CD11b+ cells. First of all, the authors should better explain the function of cxcl1 and CD11b molecules. Moreover, in order to assume that “c-Met activation may indeed control Cxcl1 expression in KCs. Thus, modulation of the c-Met-Cxcl1 axis may contribute, at least in part, to Gal-7-driven recruitment of myeloid cells to TME”, the authors should perform the analysis of transcript expression of both Cxcl1 and CD11b and also their protein abundance by western blot in co-culture in vitro system in the presence of HGF and if possible, assess the recruitment of CD11b cells by HGF containing medium.*
As explained in #14, in the revised version we made our best to clarify the link between c-Met activation and CXCL-1 expression, not only based on previous evidence from other groups but also showing new results which show: a) increased phosphorylation of c-Met in tumors, but not in normal skin, of *Tg46* compared with WT mice, and in WT compared to *Lgals7^-/-^*mice, b) a significant increase in *Cxcl1* expression by KCs upon HGF (c-Met ligand) stimulation; these results show that c-Met activation may indeed control *Cxcl1* expression in KCs; c) a trend toward an increased migration of MDSC when cultured with supernatants from HGF-treated KCs, compared to that from untreated KCs, and reduction of this effect in the presence of an anti-CXCL-1 blocking antibody. Altogether, these results suggest that CXCL1 expression dependent on c-Met activation might contribute, at least in part, to recruitment of myeloid cells to the TME.

As stated above, the impact of c-Met-dependent CXCL-1 expression on MDSC recruitment is an interesting issue which deserves a more in-depth exploration in future studies.

*17. What does it mean A.U/GAPDH in figure 4I?*

CXCL-1 expression was evaluated by real-time quantitative RT-PCR analysis in KCs upon HGF stimulation. For clarity, we have changed the y axis label to “*Cxcl1* mRNA (AU)” and the corresponding legend to be read as follows:

*“G) Cxcl1* mRNA evaluated by real time qPCR, in Kera-308 cells treated or not with 10 ng/ml HGF. **Results are presented in arbitrary units (AU) relative to Gapdh mRNA** (mean ± SEM; 3 independent experiments).”

*18. For figure 5D, E the authors should provide a representative FACS analysis as in figure 5B.*

Figure 5 was re-organized in the revised version, to avoid showing twice the same data. Thus, a unique representative FACS analysis showing MDSCs polarization upon Gal-7 binding, and its prevention in the presence of lactose was included in the revised Figure 5.

*19. Similar to other figures, the authors should align better panels and text*.

We have reviewed the typography and design details of all the figures in an attempt to provide uniformity.

*20. In the material and methods section, the authors should explain how do they use rGAL-7, and where do they obtain it*.

A detailed explanation of rGal-7 production and purification was included in the revised version (Materials and Methods section, “*Production and Purification of Recombinant Gal-7”* subsection). Also, the order of the subsections in Materials and Methods was changed, so that the production of rGal-7 precedes its use in the different assays.

Although the concentration of rGal-7 employed for each assay is detailed in each subsection, we now mentioned also these details at the end of the section: “Production and Purification of Recombinant Gal-7”, for clarity.

*21. For figure 7, if possible, the authors should show some representative images of papillomas in Gal-7-/- mice injected with either M-MDSCs-Gal-7 or M-MDSCs-Control cells.*

Representative H&E images from papillomas from *Lgals7^-/-^* mice treated either with M-MDSCs-Gal-7 or with M-MDSCs-C, as well as from *Tg46* mice treated with anti-DR5 mAb or isotype control are now included in the revised Figure 7, panels A and F.

*22. Figure7D and 7E are not clear and should be explained better. In the right panels of both figures, y axis labels are lacking. Same comment for figure 7I, J.*

For clarity, results on Figure 7D, E I and J are explained better in the revised version, as follows:

“Analysis of tumor infiltrating cells revealed a higher M-MDSC frequency (% of Ly6C^hi^Ly6G^lo^ cells from infiltrating CD11b^+^ cells; Figures 7D and S3E) and a greater percentage of Tregs (CD25^+^Foxp3/CD4^+^cells), which led to a lower CD8^+^/CD4^+^CD25^+^Foxp3^+^ ratio, indicative of attenuated T cell responses (Figure 7E and S3E) in papillomas from *Lgals7^-/-^* animals receiving M-MDSC-Gal-7 compared with those receiving M-MDSC-C.” (lines 449 to 455).

“Papillomas from anti-DR5 mAb-treated *Tg46* mice showed reduced frequency of M-MDSCs and increased proportion of PMN-MDSCs, leading to a significant drop in the M-MDSC/PMN-MDSC ratio (Figure 7I), and higher Teffector/Treg ratio (CD8^+^/CD4^+^CD25^+^Foxp3^+^ ratio), indicative of greater effector T-cell responses, compared to those treated with the control mAb (Figure 7J)”. (lines 467 to 471)

In addition, Y axis labels were included in the revised figure. We apologize for the oversight.

*23. Again, in page 19 line 108, the authors don’t introduce the function of TRAIL-R2/DR5 in the immune system. Brief sentences should be enough for readers who are not involved in the field of immunology.*

Following the reviewer´s suggestion we have included brief explanations on the mechanism of anti DR5 mAb in the revised version (Lines 135 to 138 of Materials and Methods and 459-460 of Results)

*24. Since K10 and K14 expression in skin lesions is not affected by GAL-7 function manipulation, while ki67, indicative of tumor cell proliferation, is increased upon GAL-7 hyperfunction and decreased when GAL-7 is inhibited, the authors should analyze some keratins or other skin-specific molecules and check whether these are modulated. They may start from the RNA-seq analysis for a screening.*

Following the reviewer suggestion, we have delved into RNA-seq results and analyzed the expression of additional skin-specific molecules. Despite isolated changes, no substantial alterations were observed in skin-specific transcriptomic profile, as evidenced in the heat map analysis included in the revised Supplementary Figure 1 (panel G). Noteworthy, the suprabasal-restricted keratin K1 was identified as differentially expressed in papillomas from animals with increased Gal-7 expression, and its heterodimer partner K10, also known as suprabasal marker, showed altered expression by IHC determinations (see above answer to comment #4). On the other hand, the expression of K14, which is restricted to the basal cell layer of the skin epidermis and therefore considered basal cell layer marker, remained unaltered both at mRNA (RNAseq analysis) and protein (IHC). These observations suggest that suprabasal layers´ structure rather than basal layers might be affected by Gal-7 expression. In line with these findings, Repetin, an extracellular matrix protein which expression is restricted to the differentiated suprabasal cell layers, was found to be significantly increased in papillomas from *Tg46* mice. A brief comment on this finding was included in the revised version (lines 320 to 326).

*25. The authors should explain better the functional meaning of the results described in page 19, line 420-428. When stating ”dynamic M-MDSC modulation of effector/Tregcell compartment”, the authors should briefly introduce the markers characteristic T effectors and Tregs.*

A brief explanation on these markers and ratio was included in the revised version (lines 344 to 346; see point 8.) In addition, for clarity, the above mentioned paragraph was slightly modified (lines 469 to 473)

*26. The authors should perform some molecular experiments to better characterize the-Met-Cxcl1 axis in murine and human cell lines. Most of the analyses remain exclusively correlative.*

Following the reviewer suggestion, in the revised version we show new results demonstrating that c-Met activation may indeed control *Cxcl1* expression in KCs, and that CXCL1 expression dependent on c-Met activation may contribute, at least in part, to recruitment of myeloid cells to the TME (see answer #14). We acknowledge that this topic is very interesting and deserves a more in-depth study, however, we focused our attention on the role of MDSCs in tumor development, and the impact of Gal-7 on this innate immune cell population.

**References**

1. Comoglio PM, Trusolino L and Boccaccio C. Known and novel roles of the MET oncogene in cancer, a coherent approach to targeted therapy. Nat. Rev. Cancer. 2018;18:341-358.
2. Recondo G, Che J, Jänne PA, and Awad MM. Targeting MET Dysregulation in Cancer. Cancer Discov. 2020;10:922-934.
3. Park MS, Yang AY, Lee JE, Kim SK, Roe JS, Park MS, et al. GALNT3 suppresses lung cancer by inhibiting myeloid-derived suppressor cell infiltration and angiogenesis in a TNFR and c-MET pathway-dependent manner. Cancer Lett. 2021;521:294-307.
4. Kanao, R. & Masutani, C. Regulation of DNA damage tolerance in mammalian cells by post-translational modifications of PCNA. Mutation Research/Fundamental and Molecular Mechanisms of Mutagenesis 2017; *803-805, 82–88.*

**Answers to Reviewer #2**

*The manuscript from Pinto et al describes an interesting study that reveals an essential role for Gal-7 in the initial steps of skin carcinogenesis. The authors show that elevated Gal-7 drives skin tumorigenesis in part by fostering immunosuppresive myeloid program and induces highly immunosuppresive M-MDSCs. The authors use a variety of techniques using mouse and human samples as well as RNAseq and pathway analysis. I feel their conclusions are supported by their experimental findings. Most of my issues with this study lie with the experimental design and the reporting of the experimental details. I will address them individually below but with regards to animal experiments B6 mice are normally resistant to DMBA/TPA carcinogenesis (Woodworth et al 2004 Carcinogenesis). I worry about the high doses the authors used to induce tumors in these animals as evident about* *the severe changes observed in figure 1A. Another major concern is the lack of detail in reporting mouse numbers in in vivo experiments and low mouse numbers for tumor studies. These low mouse numbers and the fact the B6 are resistent reduce confidence in the data.*

We thank the reviewer for his/her detailed analysis of our manuscript. Suggestions were of great help and were taken into consideration. New experiments were performed to address his/her concerns, which helped us to improve and strengthen our study.

We agree with the reviewer that susceptibility to DMBA/TPA carcinogenesis varies among mouse strains. Indeed, while the FVB/n strain is particularly susceptible, the C57BL/6 strain is relatively resistant to tumor formation. Despite this limitation, the use of C57BL/6 mouse strain was determined by the availability of animals either over-expressing (Tg46) or with null expression (*Lgals7^-/-^*) of Gal-7 in this background. Of note, this choice turned out to be beneficial and gave us a window of opportunity to evidence the role of Gal-7 in the development of tumors. The use of a relatively (but not absolutely) resistant strain determines a mild initial threshold which allows the effect of Gal-7 to become evident.

Regarding the doses used in DMBA/TPA carcinogenesis, we chose 200 nmol (~50ug) of 7,12-dimethylbenz[a]anthracen (DMBA) and 20 nmol (~12.5ug) of 12-O-tetradecanoyl-phorbol-13-acetate (TPA), as detailed in the Materials and methods section. Several different protocols have been described since the first description of this combination, which vary with regards to doses of the initiator or promoter to enhance or inhibit various components/effects of the protocol [1]. We have chosen the doses mentioned above, which are indeed in the range of previously described works, as listed below:

- Modi et al., 2012. Langerhans Cells Facilitate Epithelial DNA Damage and Squamous Cell Carcinoma. *Science*. 335(6064): 104–108.

The authors use 50 ug (200 nmol) DMBA and 6.25 ug TPA/week, or 100 ug (400 nmol) and 25 ug (40 nmol)/week.

- Filler et al., 2007. [Cutaneous two-stage chemical carcinogenesis.](https://pubmed.ncbi.nlm.nih.gov/21357170/) Cold Spring HarbProtocProtoc.pdb.prot4837.

The authors detail several examples of two-step carcinogenesis regimens, among them, 200 nmol (DMBA) and 10-20 nmol TPA is among the “high-dose” regimens.

- Vähätupa et al., 2019. Chemical-Induced Skin Carcinogenesis Model Using Dimethylbenz[a]Anthracene and 12-O-Tetradecanoyl Phorbol-13-Acetate

(DMBA-TPA). J. Vis. Exp154, e60445.

The authors use 200 nmol DMBA and 10 ug TPA/week

Regarding the number of animals and determinations for *in vivo* experiments and tumor studies, it was carefully reviewed and clarified in the revised version. In addition, details on the number of animals used for *in vivo* experiments are detailed below.

Please find below a point-by-point answer to your comments.

*Figure 1A-What is the justification for this dose of UVB and what was the light sourced used?*

A Waldmann UV 236 B (UV6) lamp was used for irradiation experiments, with 2.62 mW/cm^2^ intensity at the distance used (20 cm). Animals exposed from that distance during 2 min (120 seg) to this lamp received an UV dose of 0.31 J/cm^2^ (3.1 KJ/m^2^). Lamp specifications as well as the corrected UVB dose used was included in the revised version (lines 121-123). We apologize for the mistake when typing the dose (it is 3.1 instead of 13.3 kJ/m^2^).

Previous reports that uses UVB radiation to induce skin damage were considered when selecting the irradiation dose. Among them, the following ones should be highlighted:

- Lin et al., 2006. Increased Susceptibility to UV-Induced Skin Carcinogenesis in Polymerase η–deficient Mice. *Cancer Res* 66 (1): 87–94.

Mice were irradiated thrice a week in ears or dorsal back skin at a dose of 3.75 KJ/m^2^ each time.

- Lee et al., 2017. 1,8-cineole prevents UVB-induced skin carcinogenesis by targeting the aryl hydrocarbon receptor.Oncotarget. 8(62):105995-106008.

Mice were irradiated thrice a week with 1.8 to 2.0KJ/m^2^ each time**.**

- Gendronneau et al, 2015.Overexpression of Galectin-7 in Mouse Epidermis Leads to Loss of Cell Junctions and Defective Skin Repair**.** PLos One.10(3): e0119031.

Mice were exposed to a single dose of 2000J/m^2^ (2 KJ/m^2^) of UVB.

- Sha et al., 2018. Modulation of UVB-induced Carcinogenesis by Activation of Alternative DNA Repair Pathways.Sci Rep8(1):705

After a 3-week period of UVB acclimation, all mice continued to receive average weekly doses of ~22 kJ/m^2^, leading to a total cumulative exposure of ~217 kJ/m^2^

*Figure 1C-The authors state in the figure that the samples are mouse skin, yet total ear protein extracts are used. The authors should isolate dorsal mouse skin for this experiments. In addition, no details are provided about how protein extracts were prepared and the source of antibodies.*

These experiments were performed several times, firstly in ear skin, due to its easer accessibility (4 independent experiments), and thereafter in dorsal skin (3 independent experiments). Similar results were obtained with both approaches. Nevertheless, given the difficulties in isolating the epidermis from total dorsal skin samples, ear skin epidermal layer was selected for Western blot and qPCR determinations. For clarity, both options (dorsal and ear skin) are mentioned in the Materials and methods (line 194), and a brief explanation on tissue processing and on the source of antibodies is included in Supplementary Materials and methods.

*Figure 1D- A Western blot would be much more informative than IF in HaCat cells*

Following the reviewer suggestion, Gal-7 expression in response to UBV, DMBA and TPA was also evaluated by Western blot in HaCat cells. Consequently, a new panel was included in Revised Figure 1, and in Supplementary Figure 5.

*Figure 2-No description on the criteria the authors use to score tumors. In 2A is the average tumor number generated from the 5 mice per experiment or from the sum of the 4 independent experiments stated in the figure legend. This dramatically affects the confidence in the data because an N=5 is quite low for a tumor study.*

Carcinogenesis experiments corresponding to Figure 2A were performed 4 times. Each time, 3 experimental groups (*Lgals7^-/-^*, *WT* and *Tg46* mice), with 5 animals per group were evaluated. Total number of experiments, and number or animals per group in each experiment were determined following the guidelines of our Institutional Committee for the Care and Use of Laboratory Animals. Tumor scoring was developed as follows:

In each separate and independent experiment, the number of tumors in each animal was recorded at each time point, and the mean ± SD (tumors/animal) in each group was calculated.

Once all 4 experiments were executed and scored, mean ± SEM of the 4 independent experiments was calculated for each time point.

For clarity, legend to Figure 2A was modified to be read as follows:

“A) Weekly monitored tumor growth. Tumor number in each experimental group (left panel; mean ± SEM of **4 independent experiments, each experiment with n=5 mice per group**) and representative images of H&E staining of papillomas (bar represents 1000 µm) and of murine shaved backs (right panel) are shown.”

*Figure 2B the authors state Lgals7^-/-^ mice showed a lower susceptibility to skin carcinogenesis. However, the tumors per mice in 2A look similar between the WT an and Lgals7-/-. Based on the error bars these data do not seem to be in agreement.*

Figures 2 A and 2 B show different, but complementary data. While panel A shows the **number of tumors per mice** in each experimental group (average tumor number in each experimental group; mean ± SEM; n=5 per group; 4 independent experiments), panel B shows the **percentage of animals with no papillomas** (representative of 4 experiments). *Lgals7^-/-^* mice showed a lower susceptibility to carcinogenesis because at day 80 almost 50% of those animals remained tumor-free, while only 20% of Tg46 and WT animals remained free.

Panel A shows that, among *Lgals7^-/-^* mice, those animals that did develop papillomas, presented a lower number of tumors per mice. On the contrary, among *Tg46* mice, the number of tumors per mice increased along the experiment (in addition to the number of animals developing tumors)

For clarity, results in the revised version were explained as follows (lines 297 to 305):

“Interestingly, constitutive expression of Gal-7 in KCs (*Tg46* mice) resulted in a higher number of papillomas per mouse (Figure 2A) and an earlier appearance of tumors (Figure 2B), compared to *WT* and *Lgals7^-/-^*animals. At day 60, almost 80% of *Tg46* animals developed at least one tumor, compared to 50% of WT and 25% of *Lgals7^-/-^* mice (Figure 2B), with a considerably higher number of tumors per animal from day 80 onwards. On the contrary, *Lgals7^-/-^* mice showed delayed occurrence of skin lesions compared to *WT* and *Tg46* animals (Figure 2B), and consistently low or even decreasing number of papillomas per mice (Figure 2A).”

*Figure 2D-The authors state an "increase in KC proliferation rate" staining for KI67 does not measure rate, just the number of cells in the cell cycle. This wording should be changed. Measuring BrdU incorporation would provide proliferation rate.*

We agree with the reviewer that immunohistochemical detection of cell cycle-specific antigens such us Ki67 staining does not measure proliferation rate and, instead is an indicator of proliferative activity of the cells. We apologize for the mistake; wording was changed in the revised version of our manuscript to be read as follows:

“A substantial increase in the number of Ki67^+^ cells, indicative of proliferative activity, was detected (Figure 2D),…” (lines 307 to 308)

*Although B6 mice are resistant to conversion, examining how Gal-7 affects progression of the tumor would be interesting. Did the authors do any histological analysis of tumors? This could require using a different mouse strain.*

Representative images of murine shaved backs after each carcinogenesis protocol are shown in Figures 2 and 7, where differences in size, shape and number of papillomas in each strain and treatment can be appreciated. In addition, representative H&E images of papillomas from different mouse groups are shown in Figure 2, and new H&E images of *Lgals7^-/-^* mice treated either with M-MDSCs-Gal-7 or with M-MDSCs-C, as well as from *Tg46* mice treated with anti-DR5 mAb or isotype control are now included in the revised Figure 7 (panels A and F).

At the end of each protocol, papillomas were isolated and processed for additional determinations. From each tumor, a single cell suspension was prepared to determine immune cell populations by flow cytometry and another section was fixed in 4% formalin and embedded in paraffin for subsequent H&E and IHC determinations. In addition, sections were eventually processed for exome-seq and RNA-seq analysis. Though a complete histopathological assessment was not performed, macroscopic differences in lesions with enhanced Gal-7 expression, as compared to that from *Lgals7*^-/-^ mice, were consistent for every carcinogenesis experiment (see representative imagines below). Clinically apparent papillomas, typically well demarcated, symmetrical, pedunculated or dome-shaped papules without erosion or ulceration, could be appreciated in *Lgals7*^-/-^ mice, while papillomas from *Tg46* mice showed carcinoma-like appearance (poorly demarcated, asymmetrical, sessile or dome-shaped papules with erosion or ulceration). The possibility that Gal-7 may favor the transition from papilloma to carcinoma is an interesting issue that will be examined in future studies.

*The inclusion of PCNA is unclear it does more than just DNA repair.*

Proliferating cell nuclear antigen (PCNA) is an essential protein that participates in a variety of processes of DNA metabolism, including DNA replication and repair, chromatin organization and transcription and sister chromatid cohesion. In the present study, PCNA was identified as a possible Gal-7 interacting partner (through pull-down experiments followed by mass spectrometry analysis) when seeking for possible mechanisms that could eventually explain the accumulation of mutations in papillomas overexpressing Gal-7. Thus, even though PCNA has several intracellular functions as an essential factor in DNA replication and chromatin organization, we decided to explore its role in DNA repair mechanisms, that might eventually explain genomic instability.

Thus, we evaluated if any of the DNA repair mechanisms in which PCNA is known to be involved could be altered by its possible interaction with Gal-7. In our setting, neither nucleotide excision repair (NER), the main pathway used by mammals to remove bulky DNA lesions such as those formed by UV light and environmental mutagens, nor mismatch repair mechanism were affected by increased Gal-7 expression. Nevertheless, alterations in other DNA repair mechanisms, such as translesion DNA synthesis (TLS), cannot be ruled out. Indeed, post-translational modifications of PCNA play important roles in controlling DNA damage tolerance pathways, which include TLS and template switching. For example, TLS is mediated by mono-ubiquitination of PCNA while the template switching pathway is promoted by poly-ubiquitination of PCNA. Simultaneous mono-ubiquitinations on multiple units of the PCNA homotrimeric ring promote an unidentified damage tolerance mechanism that remains to be fully characterized. Furthermore, SUMOylation of PCNA in mammalian cells can negatively regulate recombination [2]. Alternatively, other PCNA functions, not related to DNA repair, might be affected by Gal-7 over expression. Moreover, the possibility that PCNA-independent mechanisms could be affected by Gal-7 overexpression cannot be ruled out. In this case, the interaction between Gal-7 and PCNA would not be related to increased number of mutations.

We acknowledge the fact that the biological relevance of PCNA-Gal-7 interaction should be further analyzed, as well as the possible implications of this molecular interaction with either DNA replication functions or with DNA repair mechanisms.

*Fig4A-The results are confusing Ccl7, Clcc1, VEGFa are highlighted as diminished in the Lgals7-/- compared to Wt and Tg46. But If Gal-7 is regulating their expression wouldn't you expect them to have higher expression in Tg46 vs wt? Only Cxcl1 follows that profile.*

We agree with the reviewer comment that only Cxcl1 follows Gal-7 expression profile. Consequently, we focused on this chemokine which is indeed involved in MDSC recruitment. For clarity, we have slightly changed the text in the revised version to be read as follows (lines 348 to 354):

“Seeking for possible mechanisms underlying the increased myeloid cell representation in the tumor microenvironment (TME) and tumor-dLN of *Tg46* mice, we examined RNAseq data, which revealed increased transcription of chemokine and cytokine genes implicated in myeloid cell recruitment in lesions from *Tg46* compared to those from WT mice (i.e. *Cxcl1* and *VEGFa*), and in papillomas from *WT* mice compared with their *KO* counterpart (i.e. *Cxcl1*, *Ccl7* and *Clcc1;* Figure 4A). Particularly interesting, *Cxcl1* expression pattern followed that of Gal-7.”

*Can the authors tie in C-Met expression with the RNAseq/pathway analysis from Figure 3.*

In the presence work, when analyzing papillomas from WT, Tg46 and *Lgals7^-/-^* mice, we detected differences in c-Met activation (p-c-Met), but not in c-Met expression levels, as evidenced in IHC analysis. Accordingly, when analyzing RNA seq data, c-Met did not belong to clusters of genes differentially expressed in distinct mouse strains (Supplementary data RNAseq). The high variability of genomic missense mutations localized particularly in c-Met gene in lesions from *Tg46* suggests that increased genomic instability might contribute as a possible mechanism implicated in altered c-Met signaling.

*Figure 4F/4G- no error bars are graphed yet there is significance noted in 4G. No detail on how many numerous were analyzed.*

Figures 4F and 4G were moved to Supplementary Figure 2 in the revised version.

Substitution types of SNVs detected across tumor types were compared using the chi-square test. Interestingly, A>G:T>C transitions were more frequently detected in Tg compared with Wt tumors (p<0.05), while A>C:T>G transversions were decreased in Tg compared with Wt tumors (p<0.05).These results were clearer explained in the revised version (lines 370 to 372) and the method used for comparisons was mentioned in the revised legend to figure S2D.

*In Figure 5- Bar graphs would be easier to read than the dot plots. It is also unclear how significance is being determined from an N=2*

Two new independent experiments, with triplicates, were performed to increase the N. In addition, following the reviewer´s suggestion, dot plots were replaced by bar graphs (Revised Figure 5).

*Figure 6. The authors state in Fig6A that there is a higher M-MDSC/PMN-MDSC ratio in Tg46 tumors that "validates in vitro findings". However there is no statically significance and based on the error bars there appears any increase would be not significant. The authors need to clarify this.*

We have modified the text in the revised version to be read as follows (lines 417 to 419):

“Although not statistically significant, results showed a trend toward a higher, M-MDSC/PMN-MDSC ratio in tumors overexpressing Gal-7 (Figure 6A).”

*Figure 7- Again I feel the experimental N is not well documented and in the case of Fig 6B an N of 6 is too small for in vivo mouse carcinogenesis studies.*

We apologize for the misunderstanding. Each carcinogenesis experiment was performed at least 3 times, with an average of 5 animals per group. Total number of experiments and number or animals per group in each experiment were determined following the guidelines of our Institutional Committee for the Care and Use of Laboratory Animals.

In particular, experiments inoculating M-MDSC-Gal-7 or M-MDSC-C weekly in *Lgals7^-/-^* mice during the two-stage carcinogenesis protocol, were performed independently three times, as detailed below

#1 *Lgals7^-/-^* mice M-MDSC-C(n=3) vs *Lgals7^-/-^* mice M-MDSC-Gal-7 (n=3)

#2 *Lgals7^-/-^* mice M-MDSC-C(n=6) vs *Lgals7^-/-^* mice M-MDSC-Gal-7 (n=6)

#3 *Lgals7^-/-^* mice M-MDSC-C(n=5) vs *Lgals7^-/-^* mice M-MDSC-Gal-7 (n=6)

Figure 6 C shows one representative experiment (#3) with 5 and 6 animals per group.

To avoid confusion, dots were deleted from bars graphs in the revised figure (panels D and E), and documentation of the number of experiments and replicates was checked in detail in the revised version.

*Figure 7F The rational of DR5 needs to be explained more. Also an N=3 is not sufficient.*

Brief explanations on the mechanism of anti DR5 mAb action and on the rational for its use were included in the revised version (Lines 135 to 138 of Materials and Methods and 459-460 of Results).

Regarding the number of experiments and replicates, each experiment was performed at least 3 times, with an average of 5-6 animals per group. In particular, *in vivo* experiments with anti DR5 mAb were performed 3 times:

#1 Tg46+iso (n=6) *vs* Tg46+anti DR5 (n=5)

#2 Tg46+iso (n=3) vs Tg46+anti DR5 (n=3)

#3 Tg46+iso (n=5) *vs* Tg46+anti DR5 (n=5)

Mean ± SEM of 3 independent experiments are shown in panels I and J. To avoid confusion, dots were deleted from bars graphs in the revised figure.

*Minor comment-
On page 12 authors refer to TPA as a carcinogen. It is a tumor promoter.*

We have changed the sentence in the revised version (lines 282-283). We apologize for the mistake.

**References**

1. Filler R., Roberts S.J., Girardi M. 2007. Cutaneous two-stage chemical carcinogenesis. Cold Spring HarbProtocProtoc.pdb.prot4837.
2. Kanao, R. & Masutani, C. 2017. Regulation of DNA damage tolerance in mammalian cells by post-translational modifications of PCNA. Mutation Research/Fundamental and Molecular Mechanisms of Mutagenesis, 803-805, 82–88.
